# Aqueous extract of *Solanum americanum* Mill. relieves functional constipation by modulating the enteric nervous system and gut micro-ecosystems

**DOI:** 10.3389/fnut.2025.1573516

**Published:** 2025-05-07

**Authors:** Xiaoyu Gao, Yanan Li, Yifan Hu, Weixing Yang, Lei Peng, Jun Sheng, Yang Tian, Lu Yao, Yan Zhao

**Affiliations:** ^1^Yunnan Key Laboratory of Precision Nutrition and Personalized Food Manufacturing, Yunnan Agricultural University, Kunming, China; ^2^College of Food Science and Technology, Yunnan Agricultural University, Kunming, China; ^3^Engineering Research Center of Development and Utilization of Food and Drug Homologous Resources, Ministry of Education, Yunnan Agricultural University, Kunming, China; ^4^Baoshan People's Hospital of Yunnan Province, Baoshan, Yunnan, China; ^5^Division of Science and Technology, Yunnan Agricultural University, Kunming, China

**Keywords:** RNA-seq, gut microbiota, neuroactive ligand-receptor interaction, TNFR1, Actinobacteriota, Christensenellaceae, Eggerthellaceae

## Abstract

*Solanum americanum* Mill. (SA) is a food and medicine homology resource of the Li nationality. In Yunnan folk, people often eat SA “for gut.” However, no studies have been reported on its treatment in constipation. The present study evaluated the laxative activity of *Solanum americanum* Mill. aqueous extract (SAAE) using loperamide-induced functional constipation (FC) mouse model. The results demonstrated that SAAE is abundant in nucleotides and polysaccharides. The gavage of 300, 600, and 900 mg/kg·bw of SAAE was efficacious in enhancing defecation behavior and the gastrointestinal transit rate of FC mice. Among these doses, 600 mg/kg·bw of SAAE exhibited the best laxative effect. Furthermore, SAAE exerted a significant effect on the colon transcriptome profiles of FC mice, most notably on the neuroactive ligand-receptor interaction and the TNF receptor type 1 (TNFR1) signaling pathway. Among neuroactive ligand-receptor interaction pathway, SAAE significantly affected the levels of 5-hydroxytryptamine, neuropeptide Y, and epinephrine in the serum and colon of FC mice. In addition, SAAE significantly up-regulated the expression of Bcl-XL, an anti-apoptotic protein in the colon. Intriguingly, SAAE also significantly increased the expression of the neuronal markers HuC/D in the colon. This finding suggests that SAAE may alleviate FC by modulating the enteric nervous system. Notably, SAAE alleviated loperamide-induced gut inflammation, damaged gut barrier, and gut microbiota disruption. A significant increase in the relative abundance of Actinobacteriota, Christensenellaceae, Eggerthellaceae, *Enterorhabdus*, and *Eubacterium_brachy_group* were observed in the gut of FC mice treated with SAAE. These microbial taxa are closely associated with phenotypic indicators of FC, and it is hypothesized that they may be key taxa in SAAE regulation of the gut micro-ecosystems and enteric nervous system to alleviate FC. These findings may contribute to the enhancement of the value and the efficient utilization of SA resources, laying a theoretical foundation for the development of laxative-related products.

## 1 Introduction

Functional constipation (FC) is a common form of constipation and its global incidence has been increasing in recent years (1, 2). FC is characterized by time-consuming and laborious defecation, decreased frequency, and discomfort after defecation, which severely impacts the life quality of patients. Numerous studies have demonstrated that the occurrence of functional constipation (FC) is closely related to dysfunction of the enteric nervous system (ENS), dysregulation of intestinal neurotransmitter secretion, impaired smooth muscle activity, and dysbiosis of the intestinal microbiota (1–3).

ENS is regarded as a distinct nervous system that can accomplish rhythmic contraction and diastole through self-regulation of intestinal smooth muscle (4). Organizational and structural changes in the ENS have been demonstrated to be a contributing factor to the development of FC. Alterations in the organizational structure of ENS can directly impair its physiological regulatory functions, manifesting as pathological features including reduced ganglion numbers with structural abnormalities, decreased neuronal density accompanied by functional impairment, and imbalance in neurotransmitter homeostasis (5). Emerging studies have further revealed that abnormal neurotransmitter levels are prevalent in the ENS of constipation patients, characterized specifically by significantly diminished concentrations of excitatory neurotransmitters and persistently elevated levels of inhibitory neurotransmitters (6). This bidirectional imbalance is recognized as one of the key pathological mechanisms underlying the development of functional constipation.

A microbial ecosystem of a highly intricate composition has been identified within the human gut. The gut microbiota plays a pivotal role in the process of nutrient absorption, immune system stimulation, and the prevention of pathogenic bacterial growth. Additionally, it produces a range of vital active compounds. Evidence suggests that alterations in the composition of gut microbiota may contribute to the development of constipation (7, 8). Several studies have shown that there is a disturbance in the composition and stability of the gut microbiota in patients suffering from constipation in comparison with healthy controls (9, 10). The microbiota of patients suffering from constipation can be characterized by a relative decrease in beneficial types of microbes, such as *Lactobacillus* and *Bifidobacterium*, and a corresponding increase in potentially pathogenic microbes, such as Proteobacteria and *Escherichia*. These changes may alter the content of bio-active compounds and the metabolic environment the intestine, thereby affecting GI motility and secretory function (10).

At this stage, the treatment of FC simply relies on laxatives, prokinetic drugs and intestinal prosecretory agents, however, these types of clinical constipation medications often suffer from the disadvantages of high toxicity, such as nausea, abdominal distension, diarrhea and drug dependence (6, 11). In recent years, natural plant-based medicinal dietary preparations have become a new direction and a new hot spot in the research of FC prevention and treatment. *Solanum americanum* Mill. (SA) is a botanical medicine of the Li nationality in China (12), and its young leaves are eaten as a vegetable for their cooling and antipyretic properties and for relieving sore throat (12–14). In Yunnan Pu'er minority areas, folk often eat the soup of SA “for gut,” and it is characterized by a high content of dietary fiber and soluble polysaccharides, so it may have laxative effect. However, there is a paucity of research on the application of SA in the management of FC and the influence of SA on GI motility. This study systematically evaluated the laxative effects of *Solanum americanum* Mill. aqueous extract (SAAE) in a loperamide-induced functional constipation (FC) mouse model. An attempt was made to elucidate the laxative mechanism from multiple perspectives, such as the ENS and intestinal microecosystems, with the help of transcriptomics and microbiomics techniques. The results may contribute to the enhancement of the medicinal and food value of SA and the efficient utilization of SA resources, and lay a theoretical foundation for the future research of related laxative products.

## 2 Materials and methods

### 2.1 Preparation of SAAE

Fresh SA (whole plant, roots removed) was harvested from the Simao district of Pu'er city, Yunnan province. The SA was then subjected to drying at 60°C and ground into a powder. Subsequently, 200 g of SA dry powder was meticulously weighed and added into boiled deionized water according to the power-water ratio of 1:9 (g/mL). The obtained mixture was then boiled for 3 min, after which it was immediately filtered using medical gauze. Following completion of the crude filtration, the filtrate was subjected to centrifugation at 4,500 rpm for 5 min, after which the upper layer was collected. The filtrate was then added into deionized water and boiled for a further 3 min, and the same operation was repeated on two more occasions. The three obtained filtrates were then combined and vacuum freeze-dried to produce dry powder of SAAE with a yield of 23%, which was refrigerated for future use. The phytochemical composition, protein and polysaccharide content of SAAE were determined by different methods (Supplementary Table S1).

### 2.2 Design of animal experiment

A total of 72 KM mice (17–22 g, male) were provided by Chengdu Dashuo Laboratory Animal Co., Ltd. and subsequently housed at 23 ± 2°C with a light/dark cyclez (12 h/12 h), with food and water provided *ad libitum*. After 7 days of acclimatization, the animals were divided into six groups of 12 mice each according to their body weights. The mice were gavaged orally once daily for a period of a week, with a volume of approximately 0.2 mL per 10 g of body weight. The blank control group (CON) was treated with saline, the constipation model group (LOP) was treated with 8 mg/kg·bw of loperamide solution, the positive control group (POS) was gavaged with 8 mg/kg·bw of loperamide and 900 mg/kg·bw of maren pills suspension, and the three administration groups were gavaged with 300, 600, and 900 mg/kg·bw of SAEE and 8 mg/kg·bw loperamide solution (The dosage was designed based on the pre-experiment results).

### 2.3 Defecation test and GI transit test

The methods of defecation test and GI transit test were previously outlined in our published literature (15). Briefly, a defecation test was preformed on the morning of the 7^th^ day of the intervention. Saline and pharmaceutical agents were administered to each group of mice following a 12 h fast. After 1 h, the mice in each group were administrated sequentially with ink, and the timing of this procedure was initiated. The first black stool time (FBST) was recorded for each mouse within 6 h. All feces excreted by each mouse were collected and enumerated in terms of fecal number (FN), wet weight (FW), and water content (FWC).

The GI transit test was preformed on the morning of day 8 of the intervention. In summary, after a 12-h fast, each group of mice was administered saline and drugs. After 30 min, ink was gavaged as an indicator. Twenty min later, the mice were euthanized, the intestines were removed, and the lengths were measured. The forward distance of the ink was measured and the GI transit rate (GTR) was calculated.

### 2.4 Enzyme-linked immunosorbent assay

Immediately following the execution of the mice, associated tissues were collected and stored at −80°C for subsequent analysis. The 5-hydroxytryptamine (5-HT) ELISA Kit (CSB-E08365m) from Wuhan Huamei Bioengineering Co., Ltd. was utilized to detect the presence of 5-HT in both serum and colon. The Epinephrine/Adrenaline (EPI) ELISA Kit (E-EL-0045) and the Mouse Neuropeptide Y (NPY) ELISA Kit (E-EL-M0820), purchased from Wuhan Elerite Biological Science and Technology Co. Ltd., were used to determinate the content of NPY and adrenaline in serum and colon, respectively. The precise operational methodologies were referenced in the instructions provided with each kit.

### 2.5 RNA extraction, reverse transcription and real-time quantitative PCR

Firstly, 20 mg of mouse colon was taken into a centrifuge tube (RNase-free) with lysate and grinding beads, and was thoroughly ground in a tissue crusher. The subsequent steps of RNA extraction were referred to the instruction manual of the TaKaRa MiniBEST Universal RNA Extraction Kit (9767, TaKaRa, Beijing). Thereafter, the concentration and purity of the eluted RNA were quantified. The subsequent steps of cDNA synthesis were referenced in the instructions provided by the PrimeScript™ RT reagent Kit (RR047A, TaKaRa, Beijing). The cDNA samples were then stored at −80°C. Finally, RT-qPCR was performed on a LightCycler480 Real-Time Fluorescence Quantification System using the TB Green^®^ Premix Ex Taq™ II (Tli RNaseH Plus) kit (RR820A, TaKaRa, Beijing). The 2^−Δ*ΔCt*^ method was employed to calibrate the relative expression of target genes, with β-actin serving as the control gene. The primers utilized are delineated in Supplementary Table S2.

### 2.6 Morphologic analysis and immunohistochemistry of colon

The fixed parts of proximal colon were fixed in 4% paraformaldehyde solution and embedded in paraffin. Colons were cut into 4 μm sections for hematoxylin and eosin (H&E) staining and Immunohistochemistry (IHC). H&E staining and IHC were performed using methods previously described method (15). Briefly, HuC polyclonal antibody (55047-1-AP, Proteintech, China), HuD polyclonal antibody (24992-1 -AP, Proteintech, China), TNFR1/CD120a polyclonal antibody (21574-1-AP, Proteintech, China), NFKB1 polyclonal antibody (14220-1-AP, Proteintech, China), a biotinylated secondary antibody (SA00001-2, Proteintech, China), and a DAB Kit (Tiangen, Beijing, China) were used in the process of IHC. The area and density of stained positive areas in IHC images, as well as the integrated optical density (IOD) of the section, were digitized using Image Pro Plus 6.0 software. Each section was assessed from six randomly selected fields.

### 2.7 Western blotting

The fixed part of proximal colon was lysed using radioimmunoprecipitation assay buffer (Strong, E121-01, Genstar, China), and the lysate was subsequently homogenized. The resulting lysate was then subjected to centrifugation at 4°C, 12,000 rpm for 3 min to remove the upper layer of oil and fat. The resultant upper layer was retained. Samples containing approximately 60 μg of protein were then loaded to 10% acrylamide gels, subjected to SDS-PAGE and transferred to a PVDF membrane (0.45 μm, Millipore). Membranes were then blocked using 5% skimmed milk powder, and incubated with Bcl-XL polyclonal antibody (10783-1-AP, Proteintech, China), and β-actin polyclonal antibody (20536-1-AP, Proteintech) overnight at 4°C. The membranes were subsequently washed using TBST buffer and incubated with HRP-conjugated goat anti-rabbit IgG (H+L) (1:10000, SA00001-2, Proteintech, China) for 1 h at 25°C. Visualization of the blots was achieved through the use of Novex™ ECL Chemiluminescent Substrate Kit (WP20005, Thermo Fisher, China). The optical density was subsequently analyzed and quantitated using Image J. The expression level of Bcl-XL was then normalized to β-actin.

### 2.8 RNA-seq of colon and bioinformatic analysis

The extraction, purification, reverse transcription, library construction, and sequencing of colonic RNA were all performed by Shanghai Majorbio Bio-pharm Biotechnology Co. The quality of the RNA was determined by a 5,300 Bioanalyzer (Agilent) and the quantity by an ND-2000 (NanoDrop Technologies). Only high quality RNA samples were used to construct sequencing libraries. The colon RNA-seq library was prepared in accordance with the Illumina^®^ Stranded mRNA Prep, Ligation (San Diego, CA) protocol, with the use of 1 μg of total RNA. The quantification of the library was performed using Qubit 4.0, and the library was then subjected to sequencing on the NovaSeq X Plus platform (PE150). The detailed construction methods are available in the Supplementary material.

Following the completion of the sequencing process, the raw sequence data statistics and quality control are conducted to ensure the integrity and quality of the subsequent analysis. The raw data undergoes a stringent quality control procedure to ensure its compatibility with the reference genome. This process is instrumental in generating data for subsequent transcript assembly, expression calculation, and related analyses. Concurrently, the quality of the comparison results of the RNA-seq is assessed. The obtained data were then subjected to a series of analytical procedures, including expression analysis, differential expression analysis, differential gene GO and KEGG annotation analysis, differential gene GO and KEGG enrichment analysis, and gene set enrichment analysis (GSEA).

### 2.9 16S rDNA sequencing and bioinformatic analysis of the cecum microbiota

The methods of 16S rDNA sequencing and bioinformatic analysis of the cecum microbiota were previously described in our published paper (15). The detailed construction methods are available in the Supplementary material. Briefly, the total gDNA of the microbiota in the mouse cecum contents was extracted by the E.Z.N.A.^®^ soil DNA kit (Omega Bio-tek, Norcross, GA, U.S.). High-quality DNA was then used as the template for PCR amplification of the 16S rRNA gene carrying Barcode sequences. The recovered products were then quantified by Qubit 4.0 (Thermo Fisher Scientific, USA). Library construction of the purified PCR products was performed using the Nextflex rapid DNA-seq kit. The samples were then subjected to sequencing on an Illumina PE300/PE250 platform.

Bioinformatic analyses of sequencing data were executed on a platform, with a focus on alpha diversity, principal coordinate analysis (PCoA), linear discriminant analysis effect size analysis, and species compositional difference analysis. Functional prediction analysis was conducted utilizing PICRUSt2.

### 2.10 Data analysis

Data were analyzed using GraphPad Prism 8.0 and the results were represented as the mean ± standard errors (SEMs). Analysis of variance (ANOVA) was used to determine significant differences between three or more groups, followed by Student-Newman-Keuls comparative test. Multivariate analysis was carried out on a cloud platform (www.majorbio.com). Binary correlation analysis was carried out using spearman method in SPSS 22.0, heat maps were drawn using HemI1.0.

## 3 Results

### 3.1 The phytochemical composition of SAAE

**Figure 1:**
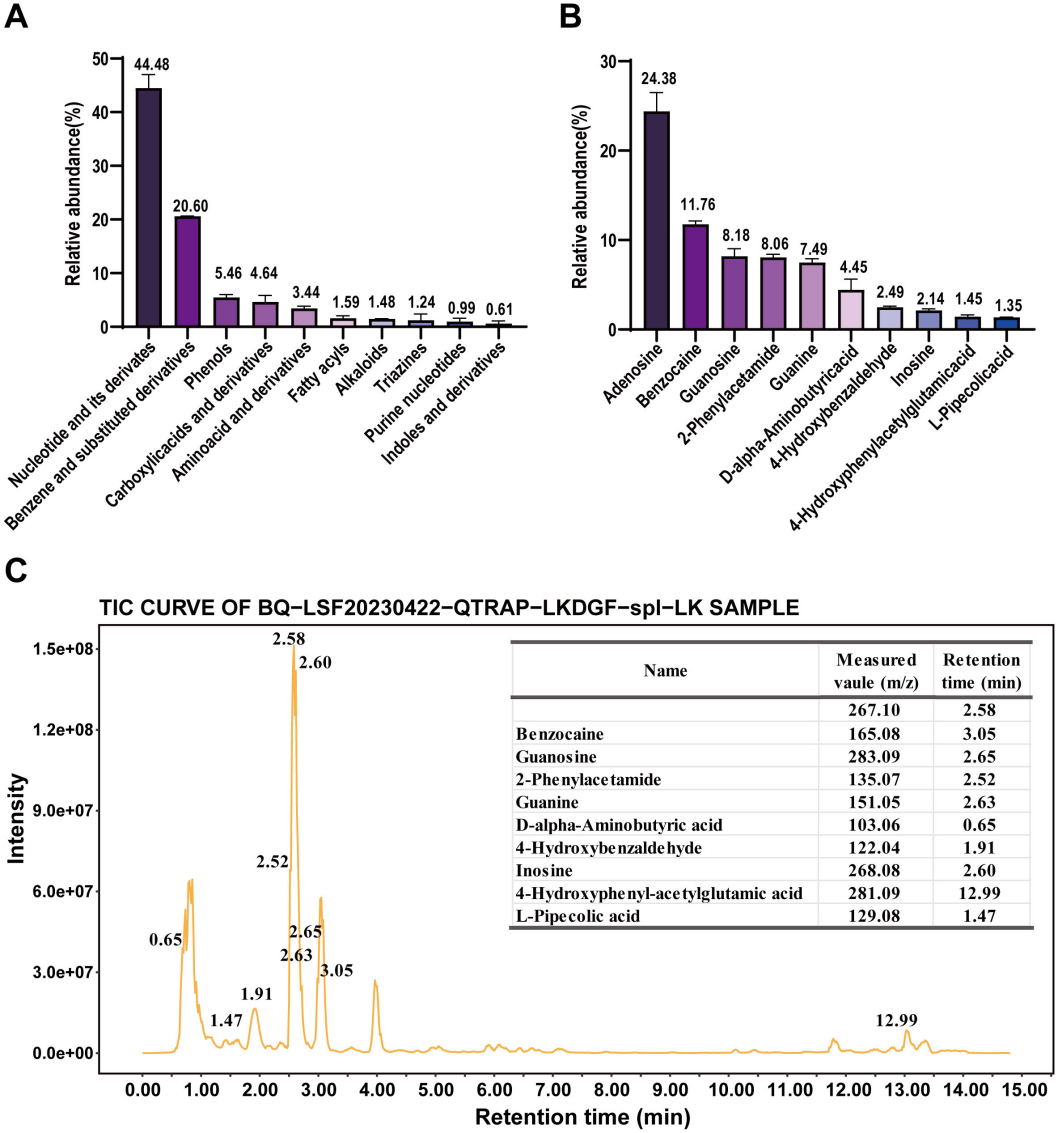
Phytochemical composition of SAAE based on an untargeted metabolomics approach for plants **(A)** main classifications of compounds in SAAE, **(B)** top 10 compounds in SAAE, **(C)** top 10 compounds in TIC plots of SAAE.

The results of untargeted metabolomics for plants (UPLC-QTRAP-MS/MS) showed that SAAE contained diverse phytochemicals, including a high relative abundance of nucleotides and their derivatives (44.48%), aromatic compounds (20.60%) and phenols and their derivatives (5.46%), as well as carboxylic acids and their derivatives, amino acids and their derivatives, fatty acyls, alkaloids, triazines, purine nucleosides and indoles and their derivatives (Figure 1A). In terms of monomeric substance composition, SAAE contains a higher relative abundance of adenosine (24.83%), ethyl p-aminobenzoate (11.68%), guanine nucleoside (8.36%), 2-phenylacetamide (8.14%), guanine (7.41%), and D-2-aminobutyric acid (4.19%), among others (Figures 1B, C, Supplementary Table S3). In addition, SAAE contained 1.10% soluble protein and 27.70% soluble polysaccharide. Overall, the high abundance of nucleotides and soluble polysaccharides in SAAE is perhaps the main active substance basis for the beneficial effects of SA on the gut.

### 3.2 SAAE relieves the loperamide-induced FC

**Figure 2:**
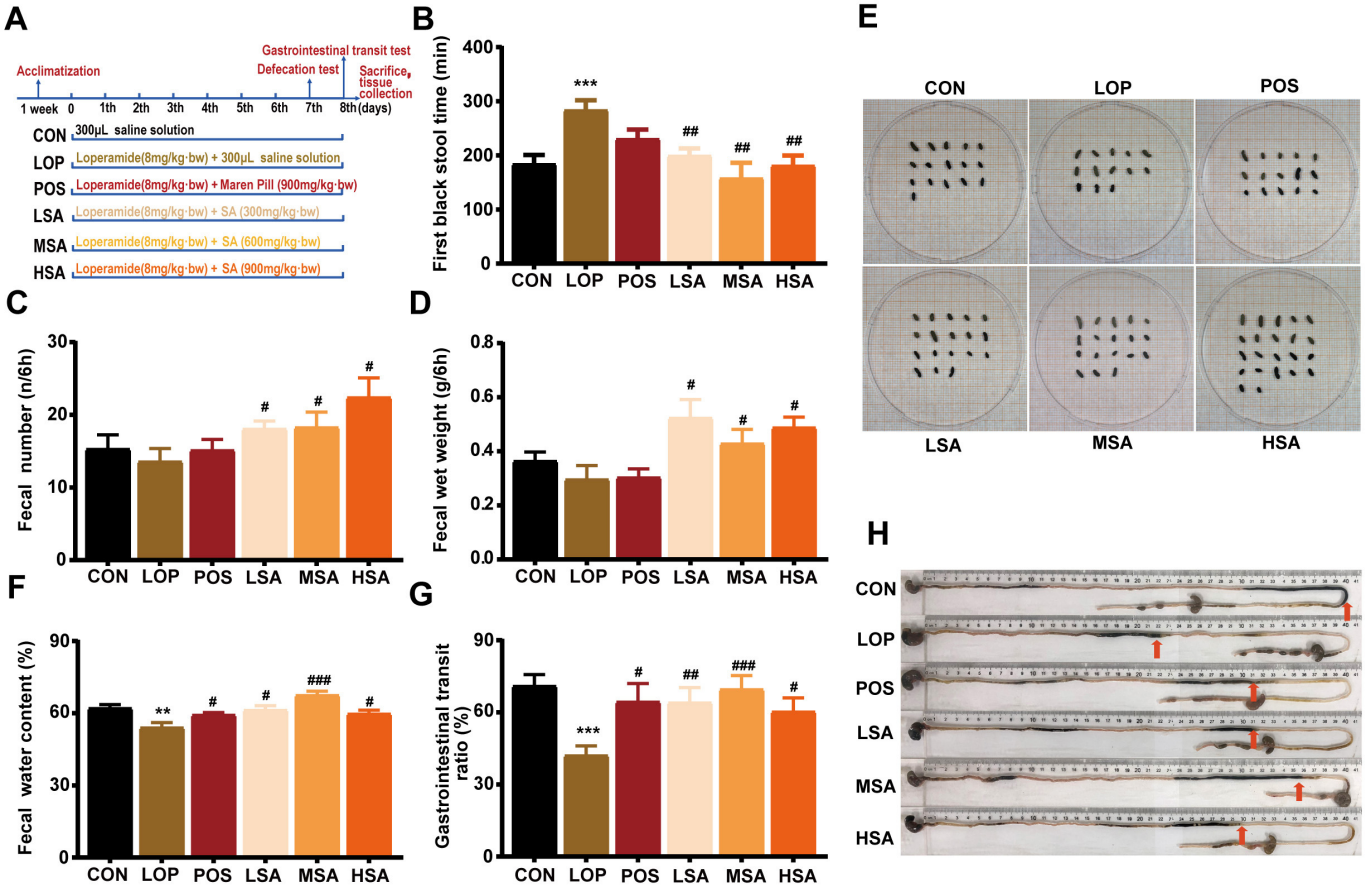
The effects of SAAE on defecation behavior and GI motility in the loperamide-induced FC mice. **(A)** The procedure and grouping for the animal experiments, **(B)** the first black stool time, **(C)** fecal number, **(D)** fecal wet weight, **(E)** representative images of the feces, **(F)** fecal water content, **(G)** GI transit rate, **(H)** representative images of GI transit. The data are expressed as the mean ± SEMs (*n* = 12). #, vs. LOP group. **, *P* < 0.01, ***, *P* < 0.001. ^#^, *P* < 0.05, ^*##*^, *P* < 0.01, ^*###*^, *P* < 0.001.

The procedure for animal testing is illustrated in Figure 2A. In the defecation test, different doses of SAAE were able to significantly reduce FBST of FC mice (Figure 2B, *P* < 0.05), increase both FN and FW during the 6 h observation period (Figures 2C, D, *P* < 0.05), and increase FWC (Figure 2F, *P* < 0.05), representative images of fecal morphology from each group are shown in Figure 2E. The medium dose of SAAE (MSA) demonstrated the best defecation-promoting effect, reducing the defecation time by 44.3% and increasing FWC by 24.9%.

In the GI transit test, the GTR of mice in the LOP group was significantly reduced compared to that of the CON group (Figures 2G, H, *P* < 0.05), different doses of SAAE significantly increased the GTR of constipated mice (*P* < 0.05), and MSA demonstrated the best GI motility-promoting effect, reducing GTR by 65.2%.

Throughout the experiment, no significant effects on body weight, food and water consumption were observed (Supplementary Figures S1A–C), no significant effects of loperamide and SAAE treatments were observed on liver, kidney and spleen indices, as well as on the small intestine length of the mice (Supplementary Figures S1D, E), but it was interesting to note that the different doses of SAAE could restore the net weight gain of the cecum caused by loperamide to some extent. Overall, SAAE relieved the symptoms of constipation and showed a good and mild laxative effect.

### 3.3 SAAE regulates the colonic transcriptome in FC mice

**Figure 3:**
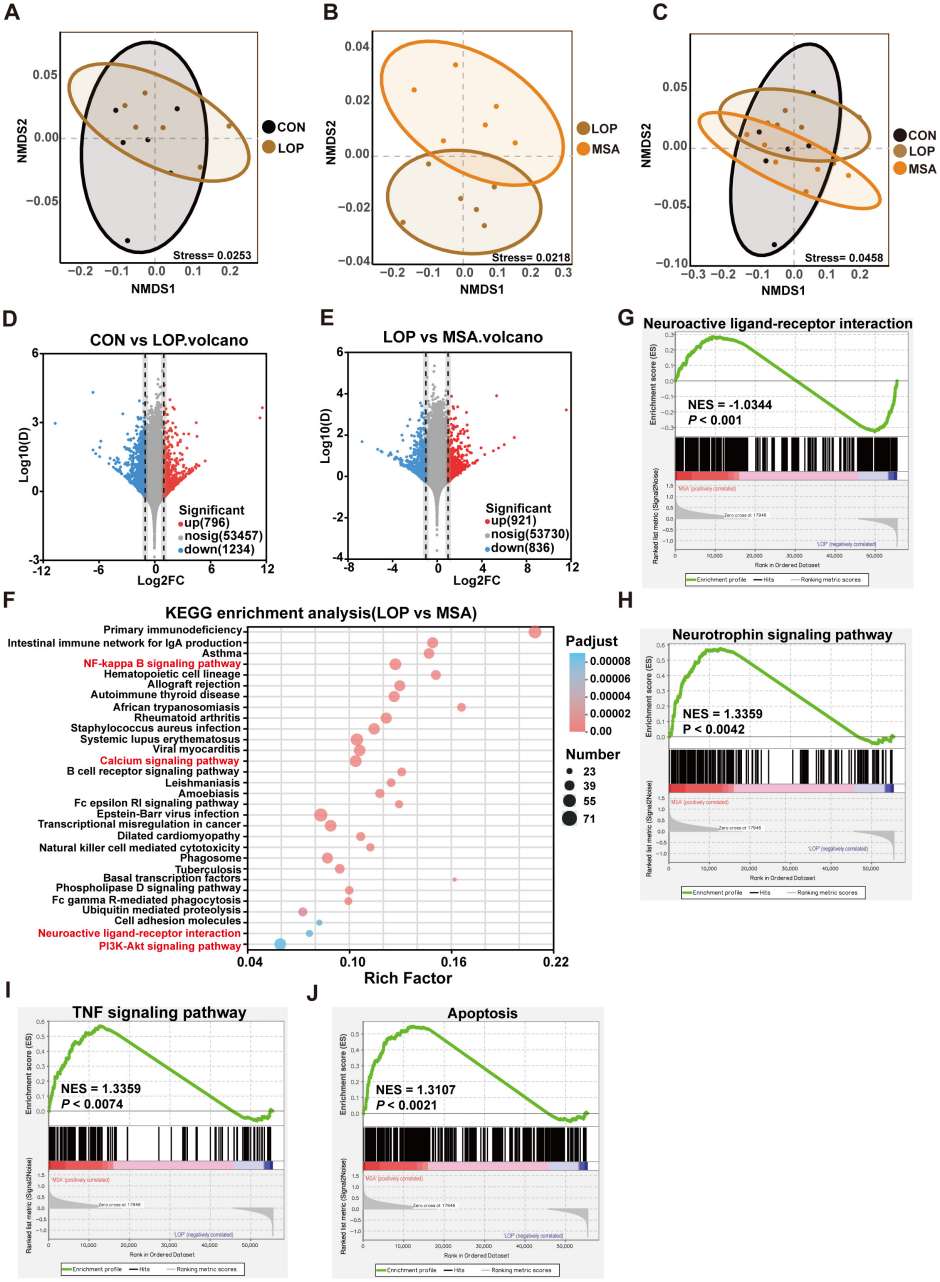
Effect of SAAE on colonic transcriptome. **(A–C)** Intergroup difference analysis based on NMDS analysis, **(D, E)** differentially expressed gene analysis based on NOIseq with threshold set at Prob > 0.8, up- and down-regulation multiplicity set at 2.0. **(F)** KEGG pathway enrichment analysis of genes in the gene set was performed using R script using Fisher's exact test, and this KEGG pathway function was considered to be significantly enriched when the corrected *P*-value (*P*_adjust_) was <0.05. The top twenty enriched pathways are shown in the figure. **(G–J)** GSEA evaluating transcriptional module of neuroactive ligand-receptor interaction, neurotrophin, TNF and apoptosis respectively between MSA treatment and LOP mice. The sequencing algorithm was Signal2Noise, with conditions set at *P*_*value*_ < 0.05, *P*_*adjust*_ < 0.25, |NES|>1. *n* = 6.

To explore the potential pathways by which SAAE ameliorates constipation symptoms in FC mice, we performed RNA-seq of mouse colonic tissues. The results of NMDS analysis showed a clear trend of separation in the sample distribution between the LOP and MSA groups, suggesting that medium-dose SAAE treatment significantly affected the colonic transcriptional profiles of FC mice (Figures 3A–C). Furthermore, the results of differential expression analysis based on the NOIseq analysis method also showed that loperamide led to the up-regulation of 796 genes and the down-regulation of 1,234 genes in the colons of the mice, compared with the LOP group, medium-dose SAAE treatment (MSA) led to the up-regulation of 921 genes and the down-regulation of 836 genes (Figures 3D, E).

GO enrichment analysis results suggested that SAAE may have a strong regulatory effect on related pathways such as immunity and inflammation in constipated mice (see Supplementary Figure S2). Furthermore, the results of KEGG enrichment analyses demonstrated that SAAE has the potential to regulate inflammation-related and immune pathways, including NF-kappa B, cell adhesion molecules, primary immunodeficiency, intestinal immune network for IgA production, and B cell receptor signaling pathway (Figure 3F). It is noteworthy that the neuroactive ligand-receptor interaction, calcium, and PI3K-Akt signaling pathway, which are intimately associated with intestinal motility, were also enriched (Figure 3F). Gene set enrichment analysis (GSEA) demonstrated that SAAE treatment significantly impacted the neuroactive ligand-receptor interaction, neurotrophin, TNF, and apoptosis. These signaling pathways, such as neuroactive ligand-receptor interactions and TNF signaling, are closely associated with intestinal motility and the pathogenesis of constipation (Figures 3G–J).

### 3.4 SAAE modulates the neuroactive ligand-receptor interaction in constipated mice

**Figure 4:**
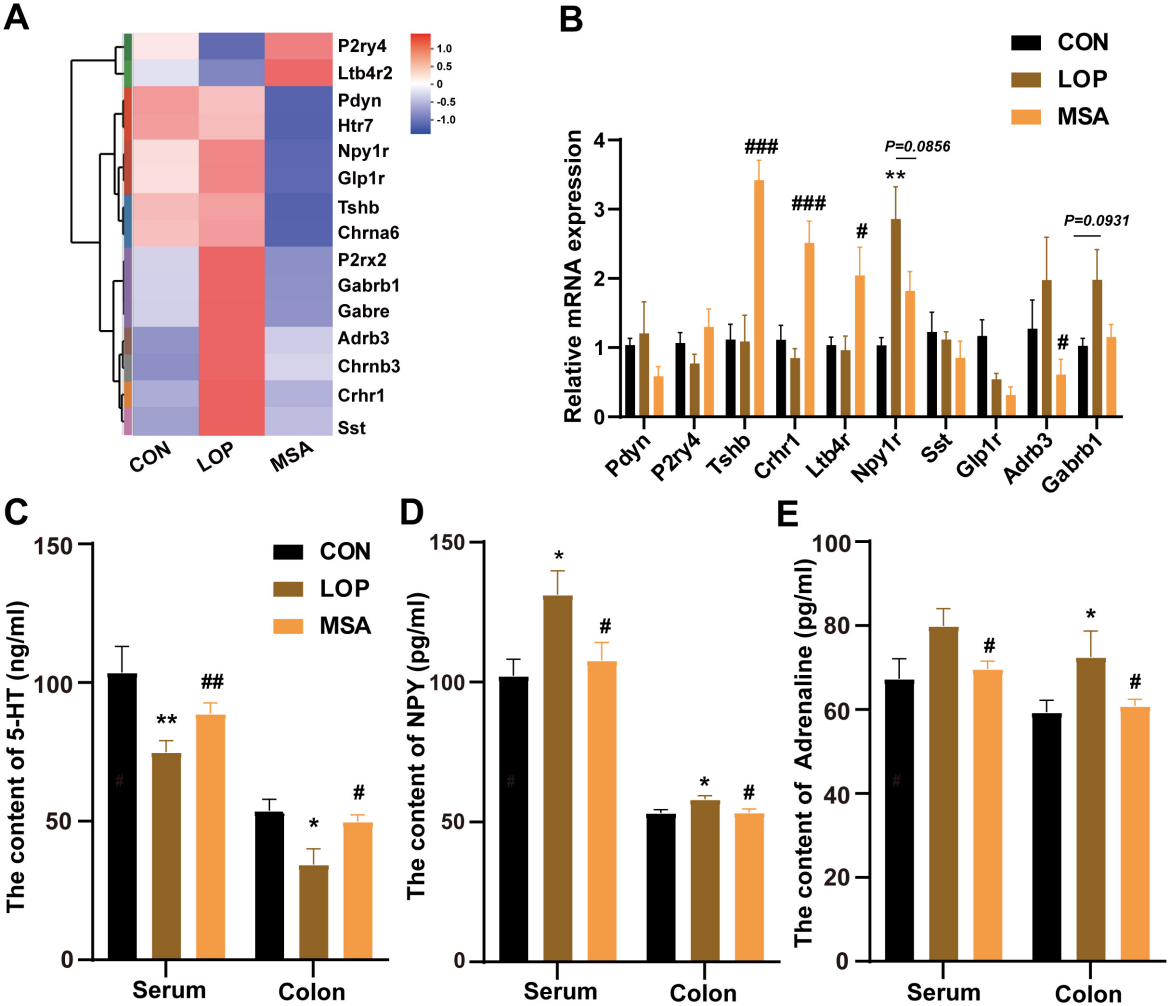
SAAE regulates neuroactive ligand-receptor interaction in the colon of FC mice. **(A)** Heatmap of the neuroactive ligand-receptor interaction (*n* = 6). **(B)** The relative expression mRNA in the neuroactive ligand-receptor interaction (*n* = 8). **(C–E)** 5-HT, NPY and Adrenaline levels in serum and colon (*n* = 8). All data presented in mean ± SEMs. *, vs. CON group, #, vs. LOP group. * *P* < 0.05, ** *P* < 0.0, ^#^ *P* < 0.05, ^*##*^ *P* <0.01, ^*###*^ *P* < 0.001.

Gene set enrichment analysis (GSEA) results suggest that SAAE has the potential to regulate the neuroactive ligand-receptor interaction in FC mice (Figure 4A). RT-qPCR was used to validate the relevant genes, and the results revealed that the expression levels of most genes in each group exhibited the expected trends (Figure 4B). Subsequent to this, the levels of 5-HT, NPY, and epinephrine (EPI) in the serum and colon of mice were examined by ELISA Kit (Figures 4C–E). The results demonstrated that loperamide induced a significant decrease in 5-HT levels in serum and colon of mice compared with the CON group, which was significantly reversed after treatment with MSA (Figure 4C, *P* < 0.05). Loperamide administration induced upregulation of both NPY and EPI levels in serum and colonic of FC mice, remarkably, MSA intervention effectively attenuated these neuroendocrine alterations, restoring concentrations to baseline levels comparable with the CON group (*P* < 0.05). These results suggest that SAAE may alleviate constipation symptoms in mice by regulating colonic neuroactive ligand-receptor interaction.

### 3.5 SAAE modulates the TNF signaling pathway and promotes colon cell survival

**Figure 5:**
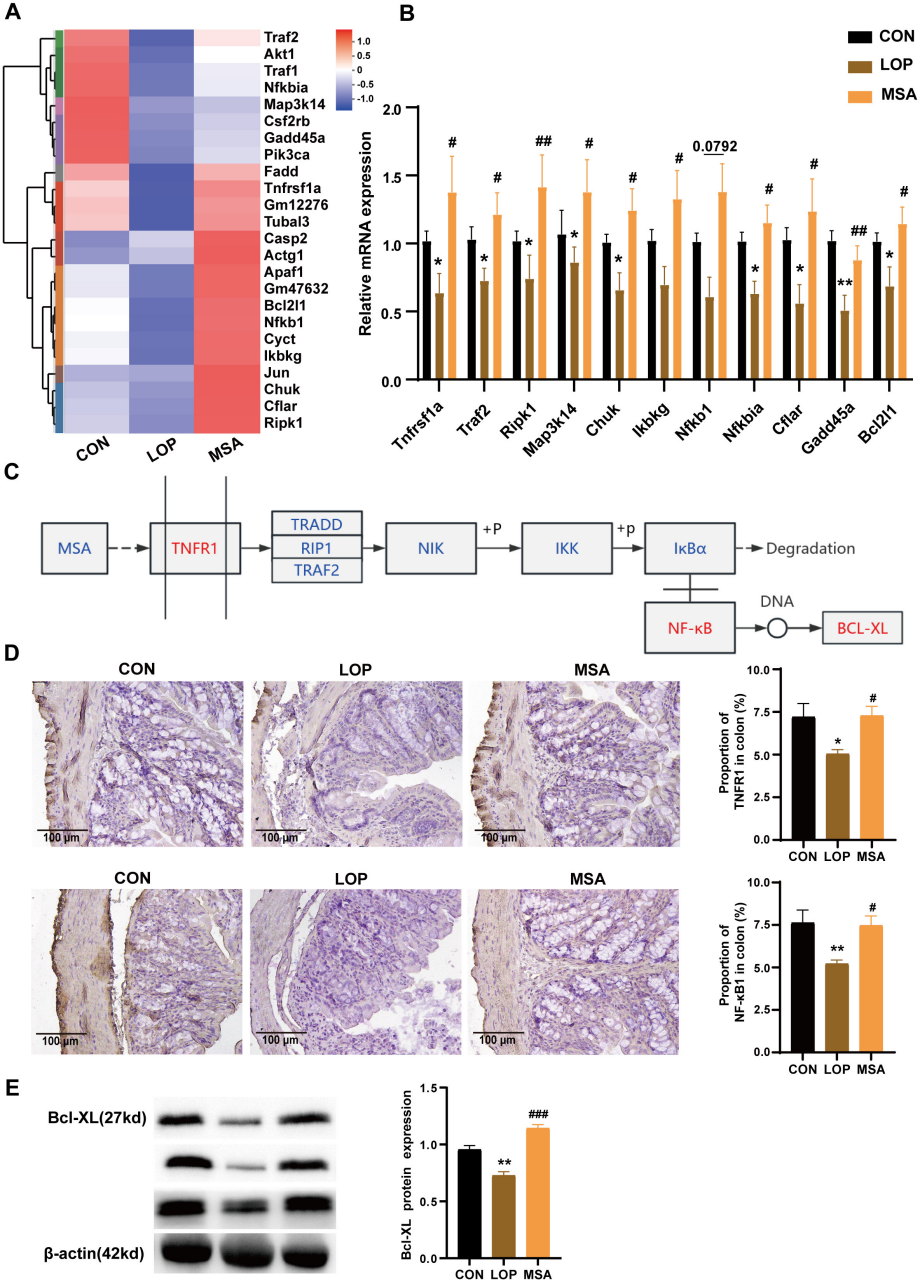
Up-regulation of colonic TNFR1 signaling pathway in FC mice by SAAE. **(A)** Heatmap of TNFR1 pathway, *n* = 6. **(B)** The relative expression mRNA in the TNFR1 pathway by RT-qPCR, *n* = 8. **(C)** Predicting the molecular mechanism by which SAAE regulates the TNFR1 signaling pathway. **(D)** Representative IHC pictures and quantitative analysis of the optical density of TNFR1 and NF-κB1 in the colon, *n* = 6. **(E)** Validation of BCL-XL protein expression, *n* = 3. All data presented in mean ± SEMs. *, vs. CON group, #, vs. LOP group. * *P* < 0.05, **P <0.01, ^#^ *P* <0.05, ^*##*^ *P* <0.01, ^*###*^ *P* <0.001.

Gene set enrichment analysis (GSEA) results of RNA-seq suggested that SAAE has the potential to upregulate the TNF signaling pathway (Figures 3H, 5A). The RT-qPCR results further validated this inference (Figure 5B). In comparison with the CON group, the colonic expression of the majority of TNF pathway genes in the LOP group was significantly down-regulated, conversely, SAAE treatment significantly increased the expression of ten related genes (*P* < 0.05), with only *Nfkb1* failing to reach the significance level (*P* = 0.0792, Figure 5B). These results are consistent with the GSEA. It was hypothesized that SAAE could promote cell survival in colon of FC mice by modulating the TNF signaling pathway (Figure 5C). To validate this hypothesis, the expression levels of TNFR1, NF-κB1, and Bcl-XL in colon were examined. The results demonstrated that loperamide significantly decreased the expression of TNFR1, NF-κB1, and Bcl-XL, while MSA treatment significantly elevated the expression of these factors (Figures 5D, E). These findings were consistent with the GSEA and RT-qPCR results. Based on this, it can be inferred that SAAE could promote cell survival in colon by modulating the TNFR1 signaling pathway.

### 3.6 SAAE increases the number of enteric neuronal cells in FC mice

**Figure 6:**
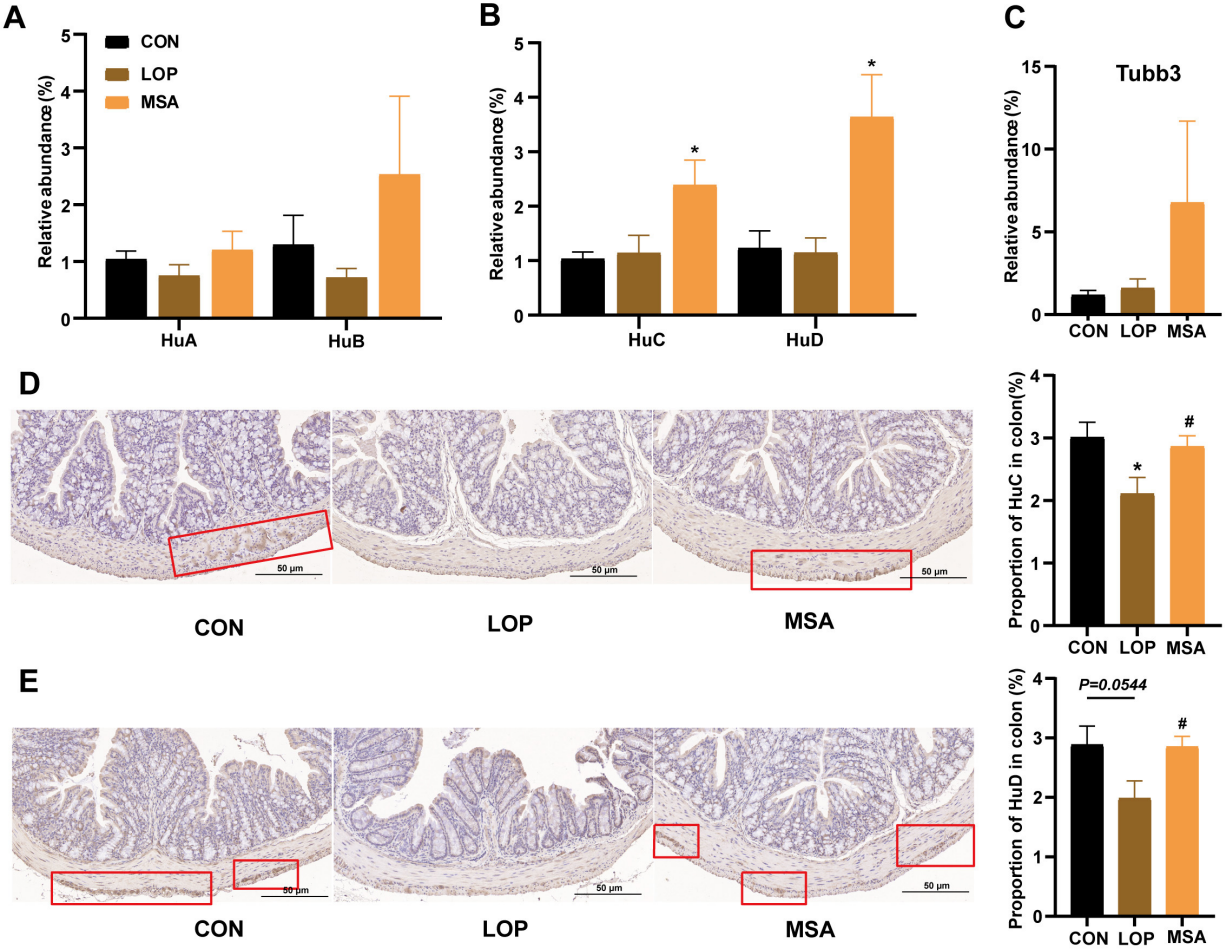
The effects of SAAE on the markers of enteric neurons in colon of FC mice. **(A–C)** The mRNA expression levels of *Hu A/B/C/D* and *Tubb3* in colon, respectively, **(D, E)** Representative IHC pictures and quantitative analysis of the optical density of Hu C and Hu D in the colon. All data presented in mean ± SEMs, *n* = 8. *, vs. CON group, #, vs. LOP group. * *P* <0.05, ^#^ *P* <0.05.

It was hypothesized that SAAE might alleviate FC by promoting the cell survival of colon that are related to GI motility. This hypothesis was based on the finding that SAAE significantly up-regulated the expression of Bcl-XL, an anti-apoptotic protein at the end of the TNF signaling pathway in the colon (Figure 5), and also significantly affected the levels of neurotransmitters in both the colon and serum (Figure 4). Enteric neuroglia, enterochromaffin cells, and enteric neurons are important units in the control of intestinal motility, and for this reason, the present study examined the expression of markers for these cells (Figure 6).

The effects of SAAE on the biomarkers of enteric neuroglia and enterochromaffin cells are shown in Supplementary Figure S3. MSA treatment elevated the mRNA expression of *Gfap, S100*β, *Plp1, Sox8*, and *Sox9*, but only significantly increased *S100*β (Supplementary Figure S3A, *P* < 0.05). Loperamide was found to induce a significant reduction of *Htr4*, whereas MSA treatment significantly restored the expression of *Htr4* (Supplementary Figure S3B, *P* < 0.05). However, MSA can not significantly enhance the relative expression of *Htr3a, Tph1*, and *Chga* (Supplementary Figure S3B).

Furthermore, SAAE differentially affected the neuronal markers in the colon (Figures 5A–C). Loperamide caused a decrease in the expression of *HuA, HuB*, and *HuD*, yet no substantial discrepancy was detected when contrasted with the CON group. In contrast, MSA treatment significantly elevated the expression of *HuC* and *HuD* (*P* < 0.05). Furthermore, immunohistochemical analysis revealed that loperamide induction led to a reduction in *HuC* and *HuD* expression, while MSA treatment resulted in a significant elevation of *HuC* and *HuD* expression (*P* < 0.05). Collectively, these observations suggest that SAAE may enhance intestinal motility by promoting the survival of intestinal neuronal cells in FC mice, thereby alleviating constipation.

### 3.7 SAAE alleviates the colonic inflammation and barrier impairment in FC mice

**Figure 7:**
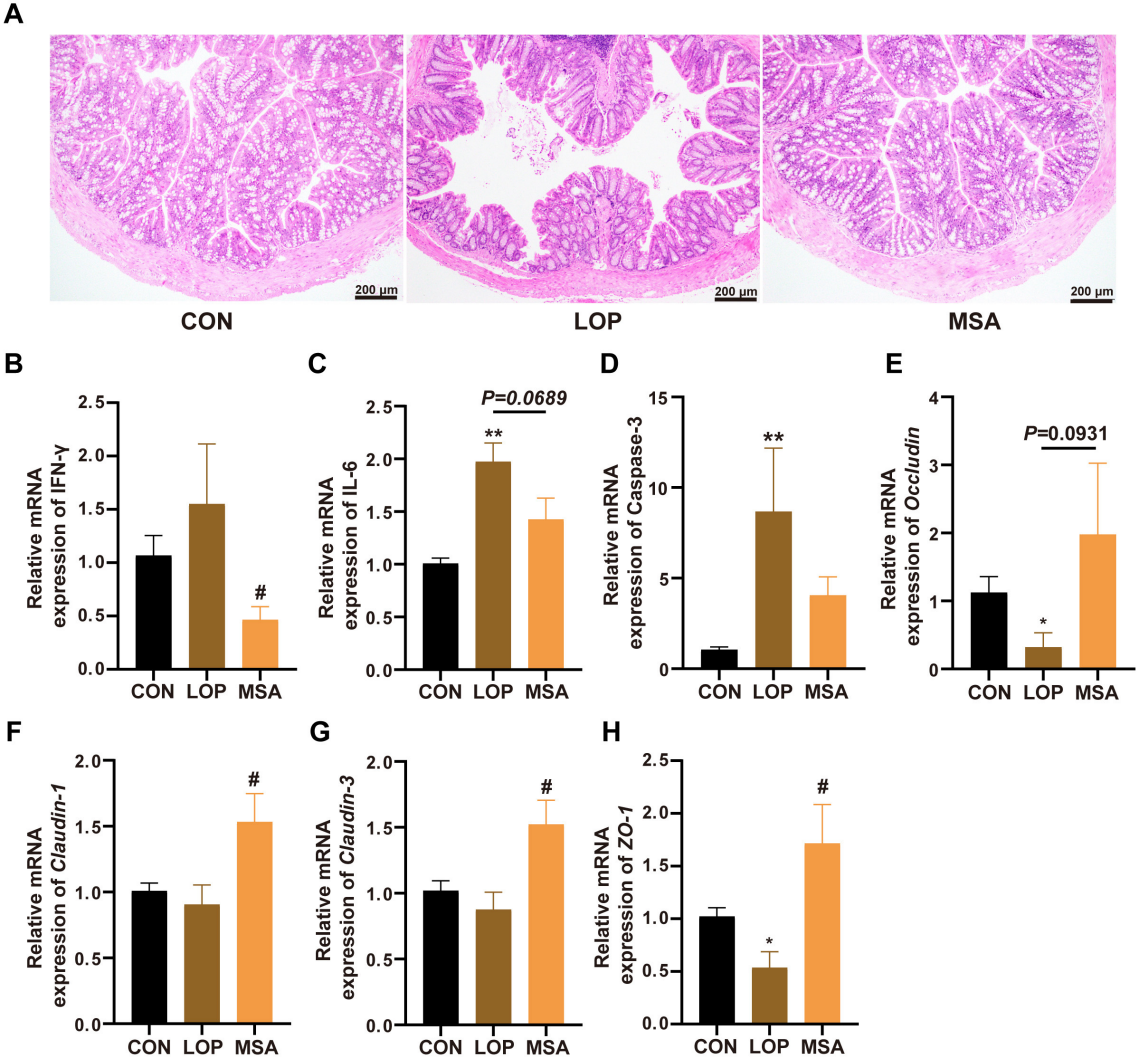
Effect of SAAE on the inflammatory status and barrier factor in colon of FC mice. **(A)** Representative photographs of H&E staining, **(B–D)** The mRNA expression levels of *INF-*γ, *IL-6*, and *Caspase-3* in colon, respectively. **(E–H)** The mRNA expression levels of *Occludin, Claudin-1, Claudin-3*, and *ZO-1* in colon, respectively. All data presented in mean ± SEMs, *n* = 8. *, vs. CON group, #, vs. LOP group. * *P* <0.05, ** *P* <0.01, ^#^ *P* <0.05.

The colon was subjected to histopathological examination using H&E staining. The intestinal villi of FC mice exhibited signs of breakage and detachment from the intestinal wall, with immune infiltration evident in the intestinal tissue. Following the intervention of SAAE, the intestinal tissue villi exhibited a neat and close alignment with the intestinal tissue of CON group (Figure 7A). The mRNA expression level of *IFN-*γ was elevated, though not significantly different to CON group. However, MSA was able to significantly reduce *IFN-*γ in FC mice (*P* < 0.05, Figure 7B). The mRNA expression levels of *Caspase-3* and *IL-6* in LOP group were higher than that in CON group, and MSA reduced *Caspase-3* and *IL-6*, but there was no significant difference (Figures 7C, D). Consequently, the present study suggests that SAAE possesses a degree of anti-inflammatory capacity, capable of effectively reducing colon inflammation in FC mice.

Furthermore, *Occludin, Claudin-1, Claudin-3*, and *ZO-1* were down-regulated by loperamide, indicating that the intestinal barrier was impaired in the FC mice. In contrast, MSA effectively prevented the damage of the gut barrier and restored the expression levels of *Claudin-1, Claudin-3*, and *ZO-1* to the level of CON group, with a significant difference (*P* < 0.05, Figures 7F–H). Therefore, it can be concluded that SAAE has a substantial capacity to alleviate loperamide-induced impairment of gut barrier function.

### 3.8 SAAE regulates the gut microbita in FC mice

**Figure 8:**
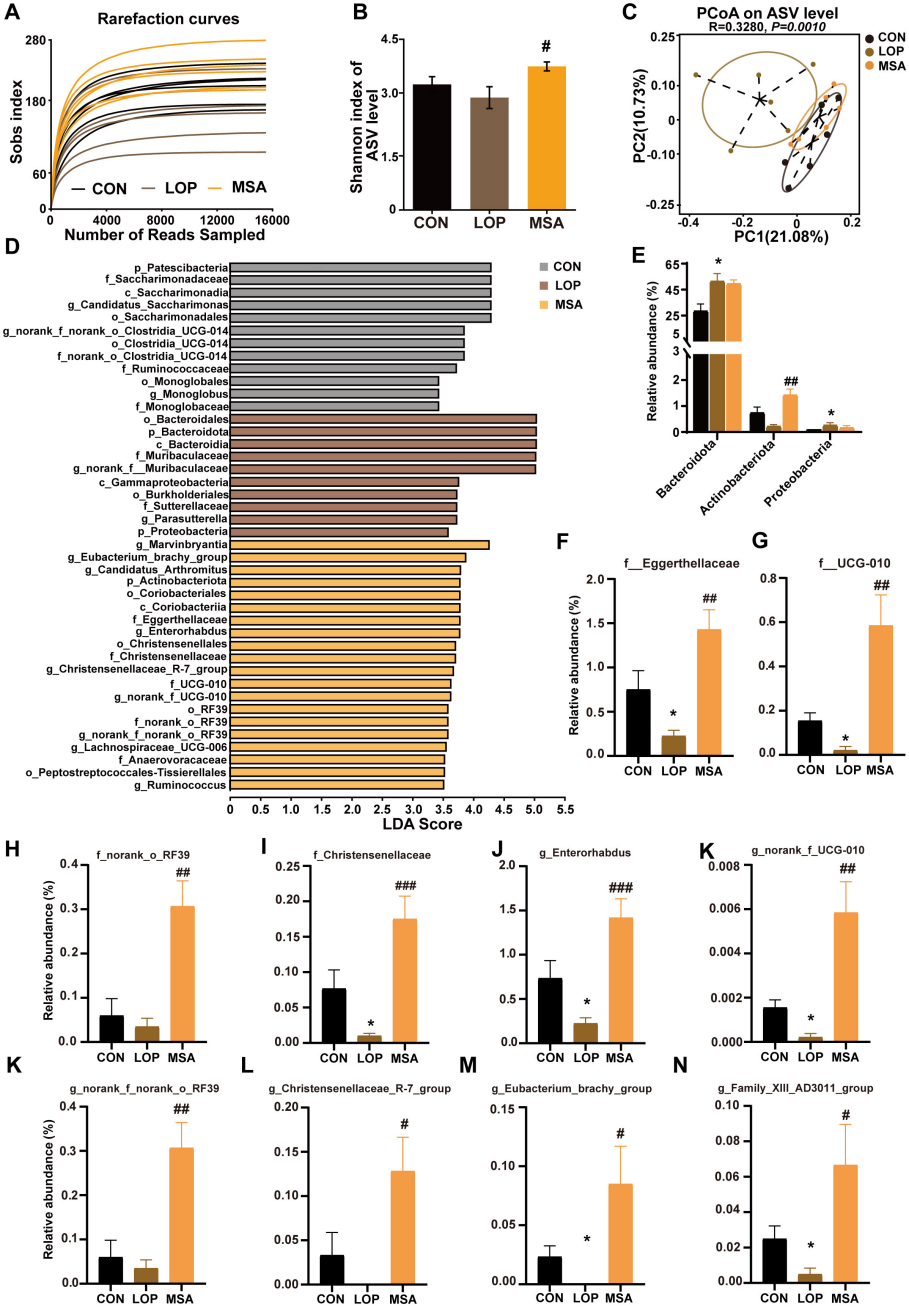
The effects of SAAE treatment on the gut microbiota structure and composition in FC mice. **(A)** Rarefaction curve, **(B)** Shannon index, **(C)** PCoA based on the unweighted unifrac distance algorithm. **(D)** LEfSe analyses (LDA score >2.0). **(E)** Relative abundance of microbes at the phylum level. **(F–I)** The relative abundances of microbes at the family level. **(J–N)** The relative abundances of microbes at the genus level. All data presented in mean ± SEMs, *n* = 6. *, vs. CON group, #, vs. LOP group. ^#^*P* < 0.05, ^*##*^*P* < 0.01, ^*###*^*P* < 0.001, *P < 0.05.

The impact of SAAE on microbial diversity in FC mice was investigated by16S rRNA gene sequencing, with the results presented in Figure 8. The Sobs index sparsity curve demonstrates a tendency to plateau with an increase in sequencing depth (Figure 8A), suggesting that the current sequencing method can reflect the diversity of the samples and yield reliable results. While loperamide exhibited a modest impact on mouse α-diversity (Figure 8A, Supplementary Figure S4), SAAE significantly augmented the diversity of cecum microbes in FC mice, as evidenced by substantial increases in Shannon, Ace, and Sobs indices. PCoA results demonstrated that β diversity of mouse cecum microbiota was influenced by loperamide, and samples of gut microbiota from SAAE-intervened mice clustered with samples from the CON group, suggesting that MSA could significantly affect the structure of gut microbial communities in FC mice (Figure 8C).

LEfSe analysis was employed to identify the dominant species in different groups at multiple levels (Figure 8D). The analysis revealed the presence of 42 distinct taxa across three distinct groups, encompassing four phyla, four orders, nine orders, 11 families, and 13 genera. Of these, the CON, ROT, and ATF groups had 12, 10, and 20 dominant taxa, respectively (Figure 8D). The focus was then placed on colonies that were significantly altered by the SAAE treatment.

At the phylum level, loperamide significantly increased the relative abundance of Bacteroidota and Proteobacteria (*P* < 0.05), and MSA reduced their relative abundance without significant differences (Figure 8E, *P*>0.05). It is noteworthy that although loperamide did not result in a significant decrease in Actinobacteriota, MSA treatment led to a significant elevation in the relative abundance of Actinobacteriota (Figure 8E, *P* < 0.05). At the family level, loperamide significantly suppressed Eggerthellaceae, UCG_010, and Christensenellaceae (*P* < 0.05), as well as norank_o_RF39, but the difference was not significant, and MSA elevated their abundances (Figures 8F–I, *P* < 0.05). At the genus level, *Enterorhabdus, norank_f_UCG_010, norank_f_norank_o_RF39, Christensenellaceae_R_7_group, Eubacterium_brachy_group*, and *Family_XIII_AD3011_group* exhibited a decrease in the LOP group, and MSA intervention significantly reversed their relative abundances (Figures 8F–I, *P* < 0.05). These results suggest that SAAE ameliorates loperamide-induced intestinal microbiota disorders in FC mice.

### 3.9 Correlations between differential microbial taxa and key host parameters

**Figure 9:**
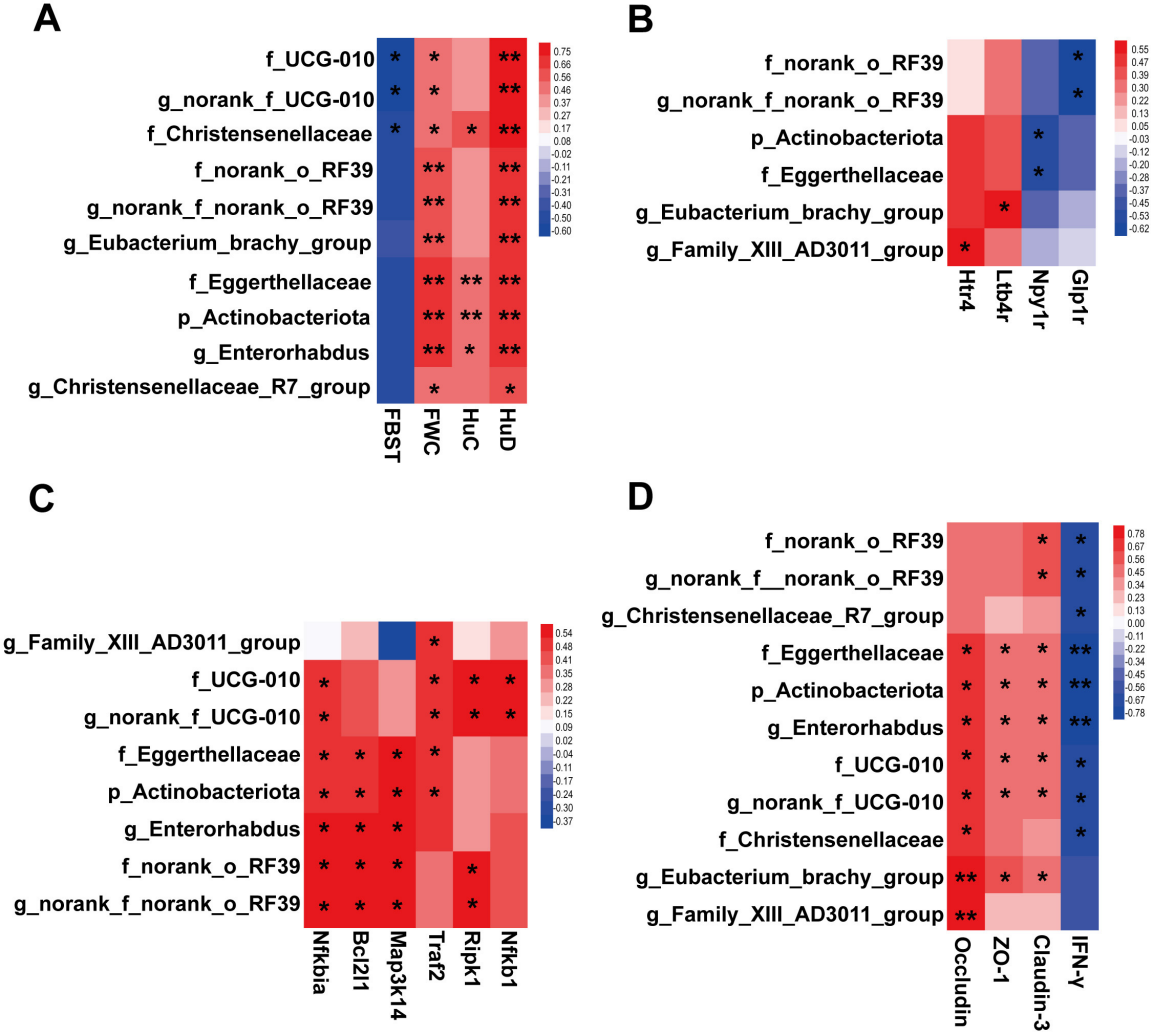
Heatmaps exhibiting correlations between microbial taxa and key host parameters. **(A)** Relationships between the microbial taxa and constipation phenotypes and neuronal markers, including FBST, FWC, *HuC* and *HuD*. **(B)** Relationships between the microbial taxa and genes in pathway of neuroactive ligand-receptor interaction. **(C)** Relationships between the microbial taxa and genes in TNFR1 signaling pathway. **(D)** Relationships between the microbial taxa and the gut inflammation and barrier factors. *Benjamini - Hochberg* (BH) programs were adopted to correct *P* values for multiple testing. * *P*_*adjust*_ < 0.05, ** *P*_*adjust*_ < 0.01.

The correlation between constipation phenotype and differential microbial taxa is demonstrated in Figure 9A. It is noteworthy that Christensenellaceae, *norank_f_UCG-010* and UCG-010 were significantly associated with both FBST and FWC. Significant positive correlations were observed between *Enterorhabdus, Christensenellaceae_R7_group*, Actinobacteriota, Eggerthellaceae, *Eubacterium_brachy_group, norank_f_norank_o_RF39*, and norank_o_RF39 with FWC. Intriguingly, all above mentioned taxa showed significant positive correlations with the expression level of the enteric neuron marker *HuD* in the colon. This finding suggests a potential for these taxa in playing important roles in modulating enteric neurons and alleviating constipation symptoms. The results of the correlation analysis also suggest a potential link between microbial and neuroactive ligand-receptor interaction pathway (Figure 9B).

As demonstrated in Figure 9C, correlations between differential microbial taxa and colonic TNFR1 signaling pathway were observed. Actinobacteriota, UCG-010, Eggerthellaceae, norank_o_RF39, *Enterorhabdus, norank_f_UCG-010*, and *norank_f_norank_o_RF39* demonstrated varying degrees of positive correlation with the mRNA expression of each factor within the TNFR1 signaling pathway. This finding suggests the potential for these taxa to modulate the TNFR1 signaling pathway. Of particular note are Eggerthellaceae, Actinobacteriota, and *Enterorhabdus*, which were found to have a significant and positive correlation with the enteric neuron marker *HuD* (Figure 9A). This suggests a complex interaction between ENS and gut microbiota under the regulation of SAAE.

Strong correlations were observed between colonic inflammatory and barrier factors and gut microbial taxa (Figure 9D). Among them, five taxa, Eggerthellaceae, Actinobacteriota, *Enterorhabdus*, UCG-010, and *norank_f_UCG-010*, were found to be strongly correlated with *IFN-*γ, *Occludin, Claudin-3*, and *ZO-1*. It is thus hypothesized that these taxa also exhibit a more important role in the amelioration of gut inflammation and barrier impairment in FC mice by SAAE.

## 4 Discussion

In current years, there has been a marked shift toward the exploration of promising alternatives from medicinal and food resources with constipation control, characterized by greater safety and efficacy. This study was the first to document the laxative effect of SA. SAAE significantly reduced the defecation time, increased fecal water content, and enhanced GI transit rate in constipated mice. However, the precise active components responsible for these laxative effects remain to be elucidated. The present study revealed the presence of soluble polysaccharides as well as a variety of phytochemicals in SAAE, with the highest relative abundance of nucleotides and their derivatives, including mainly adenosine as the major monomeric component.

A lot of research reports had published on the laxative effects of polysaccharides. For instance, *Cistanche deserticola* polysaccharides have been shown to significantly alleviate symptoms of FC and restore intestinal regulatory peptide levels, colonic pathologic damage, and colonic enteric neuronal damage (16). A substantial body of experimental research has demonstrated that nucleotides and their derivatives can stimulate the differentiation, proliferation, growth, and repair of intestinal epithelial cells (17–21), while concomitantly promoting the growth of beneficial microbes in the gut and curtailing the growth of harmful microbes, thereby reducing the likelihood of intestinal infections and injuries, and maintaining the health of the intestinal tract (22–25). However, to our knowledge, no studies have yet been published on the effects of nucleotides on intestinal motility and defecation behavior. The results in this study imply that future in-depth studies could focus on the laxative effects of polysaccharides and nucleotide to further elucidate the laxative substance basis of SAAE.

The neurotransmitter are closely related to the development of FC. The present study found that SAAE modulates the colonic neuroactive ligand-receptor interaction in the colon of FC mice, and that three neurotransmitters, 5-HT, MPY, and EPI, may be key to the relief of constipation by SAAE. 5-HT plays important roles in the control of host mood, memory, appetite, and intestinal homeostasis, and is secreted mainly by ECCs and enteric neurons (26). The production and signaling of 5-HT are vital for the development and maturation of enteric neurons. Many studies have reported reduced colonic 5-HT concentrations in patients with constipation. NPY is distributed in both central and peripheral nerves and is an important factor in neurotransmission and appetite. Research has demonstrated that the activation of TLR4 on enteric and sensory neurons could induce the production of neural NPY in large quantities in the sympathetic nerves of the gut. This occurs in conjunction with the release of norepinephrine, which in turn induces enhanced colonic relaxation and nitrosative stress (27).

Epinephrine (EPI), a hormone secreted by the body, has several types of receptors. β-adrenergic receptors are GPCRs that are existed in enteric neurons and smooth muscle cells of the gut. It has been hypothesized that β3-type adrenergic receptors are present in colonic ICCs and may modulate GI motility by inhibiting pacing potentials. Simultaneous β3-adrenergic receptor stimulation has been shown to mediate GI smooth muscle relaxation and inhibit colonic motility, thereby inducing constipation (28). Interestingly, MSA treatment down-regulated the expression levels of *Npy1r* and *Adrb3* in the colon of FC in this study, furthermore, MSA significantly down-regulated the content of NPY and EPI in serum and colon, and significantly up-regulated the serum and colon content of 5-HT. These findings suggest that MSA can improve GI dysfunction in FC mice.

Most notably, ENS transmits intestinal motility signals to different types of cells, such as enteric neuroglia, by modulating enteric neurons (29), which in turn work together to regulate intestinal motility and influence secretion and barrier function. Kulkarni et al. proposed that the rate of intestinal neuronal renewal and neurogenesis in adult mice is rapid, and that the cells of intestinal neurons often show high levels of apoptosis, but the total number of intermuscular neurons in the intestine of healthy adult mice remains essentially unchanged (30). Unlike neurons in the CNS, enteric neurons are frequently distorted, and even under normal physiological conditions, change shape in response to contractions and expansions of the musculature of the intestinal wall (31). Scholars from China found that loperamide induces a large loss of enteric neurons, and that Shouhui laxative capsule increases the expression levels of enteric neuron markers, such as Hu A/B/C/D and Tuj1, to promote enteric neuron differentiation and improve FC (32). In this study, SAAE also significantly increased the expression levels of Hu C/D, suggesting that SAAE may alleviate the symptoms of constipation by increasing the number of enteric neuron.

Bcl-XL is a well-documented anti-apoptotic protein that plays a vital role in neuronal development. The knockdown of Bcl-XL could trigger premature death in mice at the embryonic stage, with the central nervous system also exhibiting significant apoptosis. Bcl-XL has been shown to promote ATP production by mitochondria, thus ensuring the high energy demands of neurons are met and facilitating synaptic transmission (33). In this study, MSA treatment was found to up-regulate the expression of the TNFR1 pathway and the pro-survival-related protein Bcl-XL in colon, as well as increasing the expression of neuronal markers. These results suggest that SAAE can promote the survival of intestinal neurons by activating the TNF pathway to alleviate constipation.

An intact gut barrier plays a key role in keeping homeostasis. Numerous studies have demonstrated that patients experiencing constipation frequently exhibit signs of gut inflammation and impaired gut barrier. Gut inflammation is characterized by the onset of impaired intestinal barrier function, which in turn exacerbates acquired immune disorders, neutrophil and macrophage infiltration, the release of substantial quantities of cytokines and chemokines, and augmented production of pro-inflammatory factors within the intestinal epithelium. The primary components of SAAE are polysaccharides and nucleotides. Although many plant polysaccharides have improving effects on both intestinal inflammation and impaired gut barrier (7, 11, 34, 35), no studies have been reported on the relevant bio-activities of SA. In contrast, it has been demonstrated that the addition of nucleotide and nucleotide mixtures into the diet of rats results in enhanced intestinal mucosal barrier function, as evidenced by the up-regulation of ZO-1 (36). Nucleotides have been shown to reduce the incidence of small intestinal inflammation due to local ischemia by protecting small intestinal cells from free radical attack (21). In this study, SAAE treatment significantly up-regulated the colonic barrier factors, and down-regulated the pro-inflammatory factors in FC mice.

Numerous evidences support the key role of intestinal microecology in modulating intestinal motility (37). It has been confirmed that bacterial colonization of the intestine is a pivotal factor in the development and maturation of the ENS (38). Abnormal microbial composition of the GI tract may trigger perturbation of the “microbiota-gut-brain” axis, which can in turn cause altered GI motility (39, 40). Some probiotics and their biologics have favorable laxative effects (41, 42). Metabolites of gut microbes can also stimulate ENS and influence gut motility (43). The present study found that loperamide disrupted the gut homeostasis, resulting in significant changes in both the structure and diversity. Importantly, SAAE altered the cecum microbiota in loperamide-induced FC mice.

Actinobacteriota are important symbiotic taxa in the human body. Despite the absence of a definitive consensus, studies have indicated that individuals diagnosed with constipation-type irritable bowel syndrome exhibit a reduced abundance of Actinobacteria in their fecal samples when compared to healthy subjects (44). Constipation during pregnancy can lead to severe complications. The administration of probiotics has been demonstrated to effectively alleviate constipation during pregnancy, concomitantly restoring the diversity of gut microbiota, including an increase in Actinobacteria (45). Glucan-rich snail mucin heteropolysaccharide has been shown to significantly ameliorate loperamide-induced reductions in the thickness of the mucus layer and impairment of the intestinal barrier, while relieving the constipation symptoms and increasing the abundance of Actinobacteria (46). The present study also observed that Actinobacteriota was also increased by SAAE. Furthermore, constipation phenotype, intestinal neuronal markers, the TNFR1 signaling pathway, and intestinal inflammatory and barrier factors were significantly correlated with Actinobacteriota. These results provide compelling evidences to suggest that Actinobacteriota may play a significant role in the relief of constipation.

It is noteworthy that Eggerthellaceae represents a family within the phylum Actinobacteria. Extremely similar to Actinobacteriota, the present study also observed that SAAE increased the abundance of Eggerthellaceae. Furthermore, Eggerthellaceae were found to be significantly associated with many key host indicators. However, the role of Eggerthellaceae in constipation remains to be fully elucidated. A study has reported that research has shown that children with autism spectrum disorder have an increased abundance of Eggerthellaceae in their gut microbiota, which correlates with GI symptoms (47). In addition, Picrorhizae Rhizoma has been shown to relieve constipation symptoms, while concomitantly increasing the abundance of Eggerthellaceae (48). Cinnamaldehyde has been observed to attenuate *Salmonella typhimurium*-induced hepatocyte apoptosis and inflammatory response, while reversing the down-regulation of Eggerthellaceae abundance (49). These findings collectively imply a beneficial bacterial role for Eggerthellaceae expression in related diseases.

Christensenellaceae and *Christensenellaceae_R7_group* was also found to be significantly increased by SAAE. The Christensenellaceae family, a recently discovered member of the Firmicutes, is categorized as a beneficial bacterium. Approximately two-thirds of individuals diagnosed with PD also exhibit symptoms of gastric dysfunction, most notably constipation. In contrast, the relative abundance of Christensenellaceae and *Christensenellaceae_R7_group* was higher in PD patients compared to healthy controls (50–52). In this study, they were significantly associated with fecal water content, the enteric neuronal marker *HuC*, and *INF-*γ in colon. Collectively, these observations imply a potential role for Christensenellaceae in the relief of FC symptoms.

This study also revealed that *Eubacterium_brachy_group* was significantly up-regulated by SAAE in the cecum of FC mice. It is a group of gram-positive bacteria belonging to Firmicutes. No studies have been reported on its association with constipation and GI motility. However, a Mendelian randomized study assessed that *Eubacterium* was causally associated with constipation and was a safety factor, i.e., the greater the *Eubacterium* abundance, the less likely the host was to be constipated (53). The significant positive correlations between *Eubacterium_brachy_group* and fecal water content, *HuC*, and colonic barrier factors found in the present study further suggests that it may have the potential to relieve FC.

*Enterorhabdus* abundance was similarly increased by SAAE. It is a bacterium belonging to the Coriobacteriaceae family. No studies were identified in which *Enterorhabdus* was directly associated with constipation. However, a significant up-regulation of *Enterorhabdus* relative abundance was observed in studies related to constipation relief by Picrorhizae Rhizoma (48). The potential association of *Enterorhabdus* with apoptosis and neuronal regeneration is further supported by the reversal of the significant down-regulation of *Enterorhabdus* abundance by cinnamaldehyde, which also attenuated *Salmonella typhimurium*-induced hepatocellular apoptosis and inflammatory response (49). In addition, the promotion of neuronal regeneration and the attenuation of the relative abundance of *Enterorhabdus* by Chitooligosaccharides, while reducing the symptoms of mouse hepatic encephalopathy (54). The present study similarly found *Enterorhabdus* to be significantly associated with constipation phenotype, key indicators of enteric neuronal markers, TNFR1 signaling pathway, intestinal inflammation and gut barrier. These findings contribute to our understanding of the potential effect of *Enterorhabdus* in constipated mice.

## 5 Conclusion

This study provides the first experimental validation of the laxative efficacy of *Solanum americanum in vivo*, with the pioneering identification of nucleotides as novel bioactive components modulating intestinal motility and constipation relief. The potential mechanisms by which SAAE exerts its laxative effects include its ability to regulate the ENS. Specifically, SAAE not only significantly affected the colonic neuroactive ligand-receptor interaction and neurotransmitters (5-HT, NPY and EPI) in FC mice, and also protected enteric neuronal cells (HuC/D and Bcl-XL) possibly by up-regulating the TNFR1 signaling pathway to promote intestinal motility. Furthermore, SAAE has been demonstrated to possess the capacity to inhibit intestinal inflammation, repair the damaged gut barrier, and modulate the disrupted intestinal microbiota. A significant increase in the relative abundance of Actinobacteriota, Christensenellaceae, Eggerthellaceae, *Enterorhabdus*, and *Eubacterium_brachy_group* were observed in the gut of FC mice treated with SAAE. These microbial taxa are closely associated with phenotypic indicators of FC, and it is hypothesized that they may be key taxa in SAAE regulation of the ENS to alleviate FC. The findings of this study can help to improve the medicinal and edible value of SA and the effective use of SA resources, and lay a basis for the development of laxative products.

## Data Availability

The raw reads of 16S rRNA gene and RNA-seq sequence data were deposited into the NCBI Sequence Read Archive (SRA) database under BioProject accession number PRJNA1214540 and PRJNA1217329 respectively.
