# Supplementary material for: Aqueous extract of Solanum americanum Mill. relieves functional constipation by modulating the enteric nervous system and gut micro-ecosystems
Source: Front Nutr. 2025 May 7;12:1573516. doi: 10.3389/fnut.2025.1573516 (PMC12092230; doi:10.3389/fnut.2025.1573516)
Supplement: Supplementary file 1 [file Table_1.docx]

**Aqueous extract of *Solanum americanum* Mill. relieves functional constipation by modulating the enteric nervous system and gut micro-ecosystems**

**Xiaoyu Gao^1,2,3#^, Yanan Li^2#^, Yifan Hu^1,3^, Weixing Yang^2^, Lei Peng^2^,Jun Sheng^1^, Yang Tian^1^, Lu Yao^4*^,** **Yan Zhao^5*^**

1 Yunnan Key Laboratory of Precision Nutrition and Personalized Food Manufacturing, Yunnan Agricultural University, Kunming 650201, China

2 College of Food Science and Technology, Yunnan Agricultural University, Kunming 650201, China

3 Engineering Research Center of Development and Utilization of Food and Drug Homologous Resources, Ministry of Education, Yunnan Agricultural University, Kunming 650201, China;

4 Baoshan People's Hospital of Yunnan Province, Baoshan, Yunnan, 678000, China

5 Division of Science and Technology, Yunnan Agricultural University, Kunming 650201, China

# contributed equally to this work.

***** Address correspondence to Lu Yao, ylyaolyao@163.com or Yan Zhao, 2021013@ynau.edu.cn.

**Supplementary methods**

***RNA-seq of colon and bioinformatic analysis***

The total RNA was extracted from the colon using TRIzol® Reagent, following the manufacturer's instructions. The RNA quality was determined by 5300 Bioanalyser (Agilent) and the quantity was measured using the ND-2000 (NanoDrop Technologies). Only RNA samples of high quality were used to construct the sequencing library. The purification, reverse transcription, construction of the library and the process of sequencing were carried out by Shanghai Majorbio Bio-pharm Biotechnology Co., Ltd. in strict accordance with the instructions provided by the manufacturers. The colon RNA-seq library was prepared in accordance with the Illumina® Stranded mRNA Prep, Ligation (San Diego, CA) protocol, with the use of 1 μg of total RNA. Firstly, the isolation of mRNA was performed according to the polyA selection method by means of oligo (dT) beads, and the fragmentation of the RNA was subsequently induced by means of fragmentation buffer. Secondly, double-stranded cDNA was synthesized with random hexamer primers. Then, the synthesized cDNA was subjected to end-repair, phosphorylation and adapter addition according to the library construction protocol. Libraries were then size selected for cDNA target fragments of 300-400 bp, and magnetic beads were used for further processing. The libraries were then amplified by PCR for 10-15 cycles. Subsequent quantification of the library was performed using Qubit 4.0, and the library was then subjected to sequencing on the NovaSeq X Plus platform (PE150) using the NovaSeq Reagent Kit.

Following the completion of the sequencing process, the raw sequence data statistics and quality control are conducted to ensure the integrity and quality of the subsequent analysis. The raw data undergoes a stringent quality control procedure to ensure its compatibility with the reference genome. This process is instrumental in generating data for subsequent transcript assembly, expression calculation, and related analyses. Concurrently, the quality of the comparison results of the RNA-seq is assessed. The obtained data were then subjected to a series of analytical procedures, including expression analysis, differential expression analysis, differential gene GO and KEGG annotation analysis, differential gene GO and KEGG enrichment analysis, and GSEA enrichment analysis.

***16S rDNA sequencing and bioinformatic analysis of the cecum microbiota***

The total genomic DNA of the microbial community in the mouse cecum contents was extracted in accordance with the instructions of the E.Z.N.A.® soil DNA kit (Omega Bio-tek, Norcross, GA, U.S.). The quality of the extracted genomic DNA was examined by agarose gel electrophoresis using 1% agarose, and the DNA concentration and purity were determined using a NanoDrop2000 (Thermo Fisher Scientific, U.S.). High-quality DNA was then used as the template for PCR amplification of the V3-V4 variable region of the 16S rRNA gene using upstream primer 338F and downstream primer 806R carrying Barcode sequences.The PCR products were then recovered on a 2% agarose gel and purified using the DNA gel recovery and purification Kit (PCR Clean-Up Kit, China). The recovered products were then quantified by Qubit 4.0 (Thermo Fisher Scientific, USA).

Library construction of the purified PCR products was performed using the Nextflex rapid DNA-seq kit.The samples were then subjected to sequencing on an Illumina PE300/PE250 platform. The quality control of the double-ended raw sequencing sequences was performed using the fastp (https://github.com/OpenGene/fastp, version 0.19.6) software and the FLASH (http://www.cbcb.umd.edu/software/flash, version 1.2.11) software was used for splicing. The noise-reduced optimized sequences obtained after QC splicing were then processed using the DADA2 plugin (or Deblur plugin) in the Qiime2 process to obtain ASVs. The ASVs were then analysed for species taxonomy based on the Silva 16S rRNA gene database (v 138) using the Naive Bayes classifier in Qiime2. Bioinformatic analyses of sequencing data were executed on the cloud platform (https://cloud.majorbio.com), with a focus on alpha diversity, Principal coordinate analysis(PCoA), Linear discriminant analysis effect size analysis (LEfSe) and species compositional difference analysis. 16S functional prediction analysis was conducted utilizing PICRUSt2 (version 2.2.0) software.

**Supplementary tables and figures**

**Table S1 The main nutritional components of *Solanum americanum* aqueous extract (SAAE)**

| Composition | Content (%) | Method | Reference |
| --- | --- | --- | --- |
| Protein | 1.10 | Kjeldahl | [1] |
| Polysaccharide | 27.70 | Spectrophotometry | [2] |
| Phytochemical composition | - | HPLC-QTRAP-MS/MS | [3] |

**Table S2 Primers sequences used for quantitative PCR analysis**

| Gene | Primer | Sequence（5´to 3´） |
| --- | --- | --- |
| *Pdyn* | Forward | CTCCTCGTGATGCCCTCTAAT |
|  | Reverse | AGGGAGCAAATCAGGGGGT |
| *P2ry4* | Forward | ATGACCAGTGCAGACTCCTTG |
|  | Reverse | GAGGCAACAGGATGAACTTGA |
| *Ltb4r2* | Forward | ATGTCTGTCTGCTACCGTCC |
|  | Reverse | AGCTCCATACTACGAAGCCAT |
| *Npy1r* | Forward | TGATCTCCACCTGCGTCAAC |
|  | Reverse | ATGGCTATGGTCTCGTAGTCAT |
| *Gabrb1* | Forward | TCCCGTGATGGTTGCTATGG |
|  | Reverse | CCGCAAGCGAATGTCATATCC |
| *Adrb3* | Forward | GGCCCTCTCTAGTTCCCAG |
|  | Reverse | TAGCCATCAAACCTGTTGAGC |
| *Tnfrsf1a* | Forward | CCGGGAGAAGAGGGATAGCTT |
|  | Reverse | TCGGACAGTCACTCACCAAGT |
| *Bcl2l1* | Forward | GACAAGGAGATGCAGGTATTGG |
|  | Reverse | TCCCGTAGAGATCCACAAAAGT |
| *Cflar* | Forward | GCTCCAGAATGGGCGAAGTAA |
|  | Reverse | ACGGATGTGCGGAGGTAAAAA |
| *Gadd45a* | Forward | CCGAAAGGATGGACACGGTG |
|  | Reverse | TTATCGGGGTCTACGTTGAGC |
| *Traf2* | Forward | AGAGAGTAGTTCGGCCTTTCC |
|  | Reverse | GTGCATCCATCATTGGGACAG |
| *Ripk1* | Forward | GAAGACAGACCTAGACAGCGG |
|  | Reverse | CCAGTAGCTTCACCACTCGAC |
| *Map3k14* | Forward | TGTGGGAAGTGGGAGATCCTA |
|  | Reverse | GGCTGAACTCTTGGCTATTCTCA |
| *Chuk* | Forward | GTCAGGACCGTGTTCTCAAGG |
|  | Reverse | GCTTCTTTGATGTTACTGAGGGC |
| *Nfkbia* | Forward | ATGGCAGACGATGATCCCTAC |
|  | Reverse | TGTTGACAGTGGTATTTCTGGTG |
| *β-actin* | Forward | GGCTGTATTCCCCTCCATCG |
|  | Reverse | GGCTGTATTCCCCTCCATCG |

**Table S3 Chemical compounds of SAAE (Relative abundance >0.5%)**

| Name | CAS | Formula | Relative abundance (%) |
| --- | --- | --- | --- |
| Adenosine | 58-61-7 | C_10_H_13_N_5_O_4_ | 24.38 |
| Benzocaine | 94-09-7 | C_9_H_11_NO_2_ | 11.77 |
| Guanosine | 118-00-3 | C_10_H_13_N_5_O_5_ | 8.18 |
| 2-Phenylacetamide | 103-81-1 | C_8_H_9_NO | 8.06 |
| Guanine | 73-40-5 | C_5_H_5_N_5_O | 7.49 |
| D-alpha-Aminobutyric acid | 2623-91-8 | C_4_H_9_NO_2_ | 4.45 |
| 4-Hydroxybenzaldehyde | 123-08-0 | C_7_H_6_O_2_ | 2.49 |
| Inosine | 58-63-9 | C_10_H_12_N_4_O_5_ | 2.14 |
| 4-Hydroxyphenylacetylglutamic acid | - | C_13_H_15_NO_6_ | 1.45 |
| L-Pipecolic acid | 3105-95-1 | C_6_H_11_NO_2_ | 1.35 |
| Deethylatrazine | 6190-65-4 | C_6_H_10_ClN_5_ | 1.24 |
| Phloretic acid | 501-97-3 | C_9_H_10_O_3_ | 1.02 |
| Adenosine 2',3'-cyclic phosphate | 634-01-5 | C_10_H_12_N_5_O_6_P | 0.99 |
| 2-Picolinic acid | 14639-25-9 | C_6_H_5_NO_2_ | 0.86 |
| 5-Aminovaleric acid | 660-88-8 | C_5_H_11_NO_2_ | 0.72 |
| Riboflavine | 83-88-5 | C_17_H_20_N_4_O_6_ | 0.58 |
| Xanthosine | 146-80-5 | C_10_H_12_N_4_O_6_ | 0.57 |
| Leukoaminochrome | 29539-03-5 | C_8_H_9_NO_2_ | 0.57 |
| L-Tyrosine | 60-18-4 | C_9_H_11_NO_3_ | 0.57 |
| Miltirone | 27210-57-7 | C_19_H_22_O_2_ | 0.56 |
| N,N-Dimethyl-1,4-phenylenediamine | 99-98-9 | C_8_H_12_N_2_ | 0.54 |
| Oleic acid | 112-80-1 | C_18_H_34_O_2_ | 0.53 |
| Salicylic acid | 69-72-7 | C_7_H_6_O_3_ | 0.51 |

**
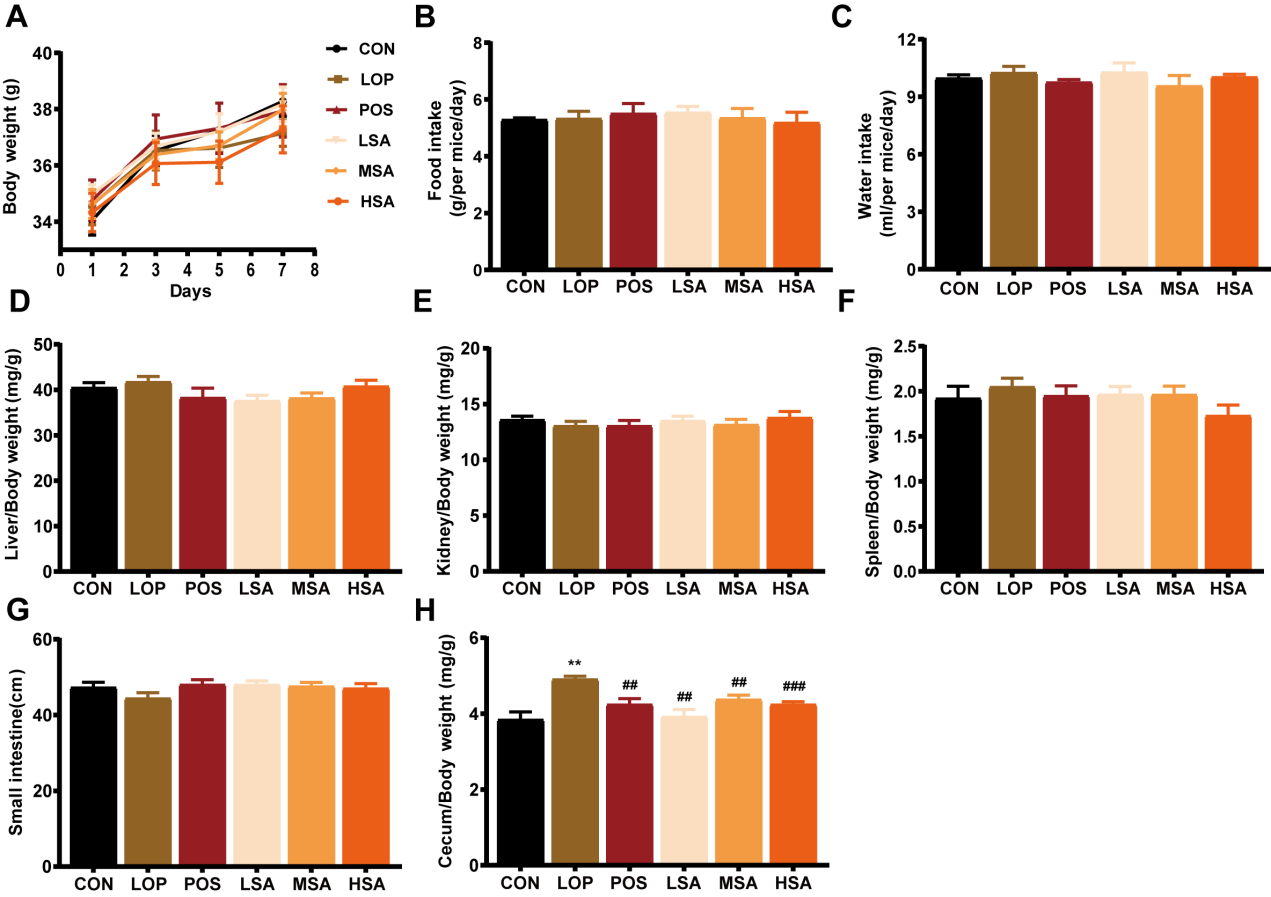
**

**Fig. S1 The effect of SAAE on body weight, food and water intake, and visceral Workflow of animal experiments.**

1. Dynamic changes of body weight; (B-C) Average food and water intake; (D) Liver index; (E) Kidney index; (F) spleen index; (G) The average length of small intestine; (H) Cecum index. All data are presented in the form of means ± SEM, *n* = 12. All data are presented in the form of means ± SEM, *n* = 12. *, vs. CON group; ** *P*<0.01; #, vs. LOP group. ## *P*<0.01, ### *P*<0.001.

**
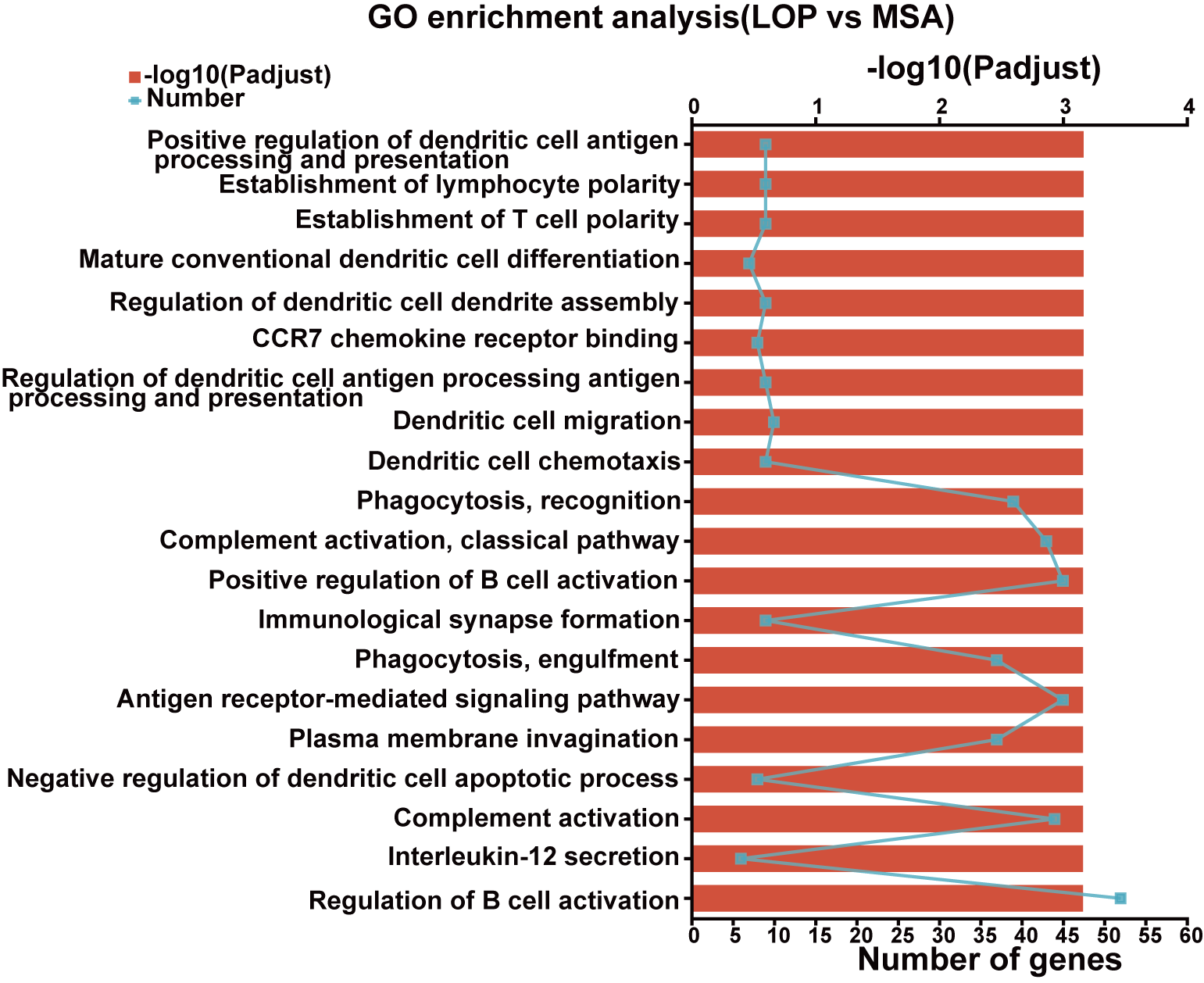
**

**Fig. S2 GO functional enrichment analysis based on the deferentially expressed genes**

Genes in the gene set were analyzed for GO enrichment using the software Goatools using Fisher's exact test, and this GO function was considered to be significantly enriched when *P*_adjust_ < 0.05. The differential gene set was derived from the NOIseq-based differential analysis method with the threshold set at *Prob*>0.8 and the up- and down-regulation multiplicity set at 2.0.

**
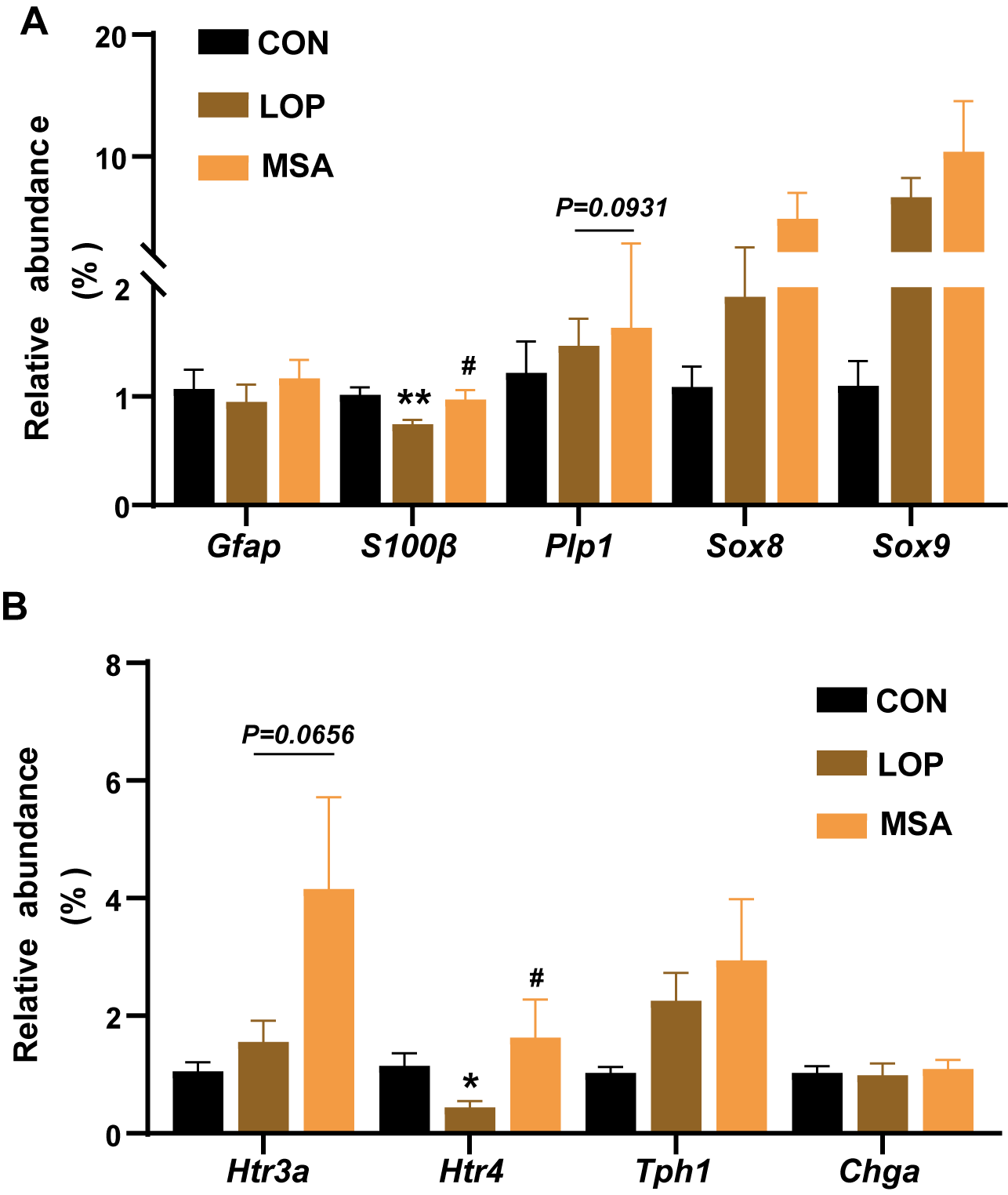
**

**Fig. S3 The effects of SAAE on the markers of enteric glial cells and enterochromaffin cells in colon of FC mice**

(A) The mRNA expression of enteric glial cell markers; (B) The mRNA expression of in enterochromaffin cell markers. All data presented in mean ± SEMs, *n* = 8. *compared with the CON group; # compared with the LOP group. * *P* <0.05, ** *P* <0.01, # *P* <0.05.

**
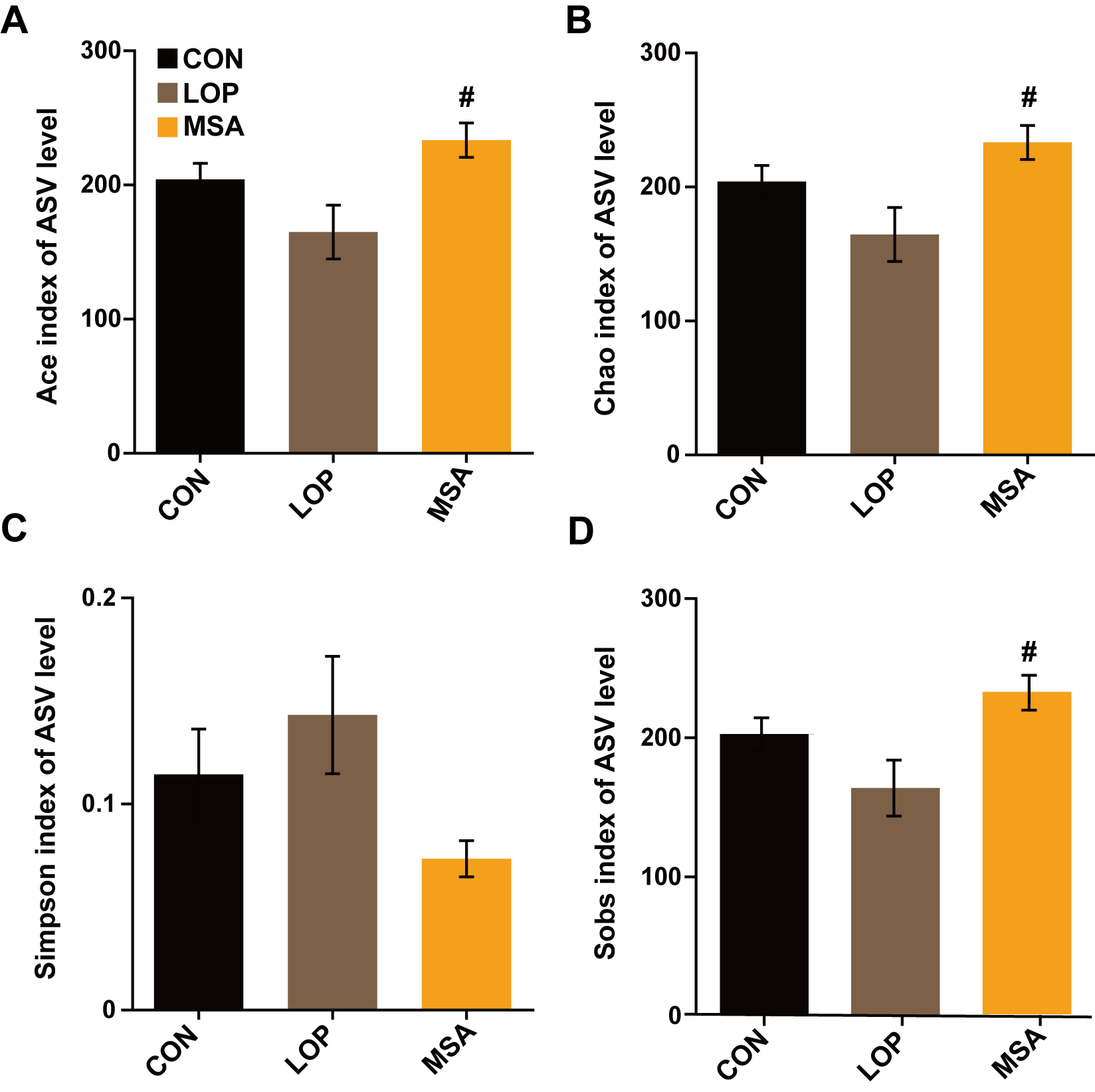
**

**Fig. S4 The effect of SAAE on α-diversity indices of cecal microbiota in FC mice**

**References:**

[1] GB 5009.5-2016, National food safety standard-Ddetermination of protein in food[S]. (In Chinese)

[2] NY/T 1676-2008, Determination of crude polysaccharide in edible fungi[S]. (In Chinese)

[3] Doppler, M., Kluger, B., Bueschl, C., Schneider, C., Krska, R., Delcambre, S., Hiller, K., Lemmens, M., & Schuhmacher, R. (2016). Stable Isotope-Assisted Evaluation of Different Extraction Solvents for Untargeted Metabolomics of Plants. International journal of molecular sciences, 17(7), 1017. https://doi.org/10.3390/ijms17071017.
